# Formation control between leader and migratory follower tissues allows coordinated growth

**DOI:** 10.1126/sciadv.ads2310

**Published:** 2025-07-30

**Authors:** Toru Kawanishi, Takamichi Sushida, Tony Y.-C. Tsai, Hiroyuki Takeda, Sean G. Megason

**Affiliations:** ^1^ Department of Systems Biology, Harvard Medical School, Boston, MA 02115, USA.; ^2^ Department of Biological Sciences, Graduate School of Science, University of Tokyo, Tokyo 113-0033, Japan.; ^3^ School of Life Science and Technology, Institute of Science, Tokyo, Kanagawa 226-8501, Japan.; ^4^ Faculty of Informatics, University of Fukuchiyama, Kyoto 620-0886, Japan.; ^5^ Department of Developmental Biology, Washington University School of Medicine, St. Louis, MO 63110, USA.

## Abstract

Coordinated growth of multiple tissues is fundamental to shaping our body, but the underlying mechanisms remain underexplored. In zebrafish embryos, midline tissues composed of the notochord, floorplate, and hypochord elongate synchronously with their lengths aligned. We show that floorplate and hypochord cells collectively migrate posteriorly along the nascent notochord extracellular matrix as it extends posteriorly, maintaining the tripartite configuration. Fibroblast growth factor-mediated migration in a spatially graded manner causes cell stretching, which triggers Yap-dependent proliferation and controls floorplate and hypochord growth. Supported by mathematical modeling, we further suggest that their growth is fine-tuned by mechanical tethering to the notochord via cadherin 2 at the posterior end. We propose that the notochord instructs and sustains the tripartite formation via leader-follower formation control, a strategy from engineering that spatially organizes multiple agents to coordinate the growth of the midline tissues.

## Teaser

Zebrafish midline tissues coordinately elongate via leader-follower formation control.

## INTRODUCTION

The animal body is a complex structure composed of multiple tissues precisely arranged in three-dimensional (3D) space to ensure cohesive functionality. During development, different types of tissues, such as muscles, blood vessels, and nerves, grow concomitantly and integrate to form structures like the limbs, heart, and trunk (*1*–*5*). While the initial steps often entail the differentiation and formation of each tissue independently of one another, the overall organization of these structures exhibits complete alignment of their dimensions, indicating the necessity of cellular communication between tissues. Despite recent advances in understanding the cellular and molecular machineries underlying the growth of individual tissues during development (*6*, *7*), the mechanisms underlying the choreography of such coordinated tissue growth remain largely elusive.

Control theory is a field of engineering dedicated to regulating the behavior of dynamic systems to achieve desired outputs and has proven to be a useful approach for understanding how complex systems maintain robustness against variations in initial conditions and inherent noise within the processes (*8*–*10*). An emerging branch of control theory, known as “formation control,” addresses how the spatial arrangement of multiagent collectives, such as flocks of animals, swarms of robots, or collections of drones, can be maintained (*11*–*13*). However, this theory has not yet been applied to multicellular systems.

**Fig. 1.**
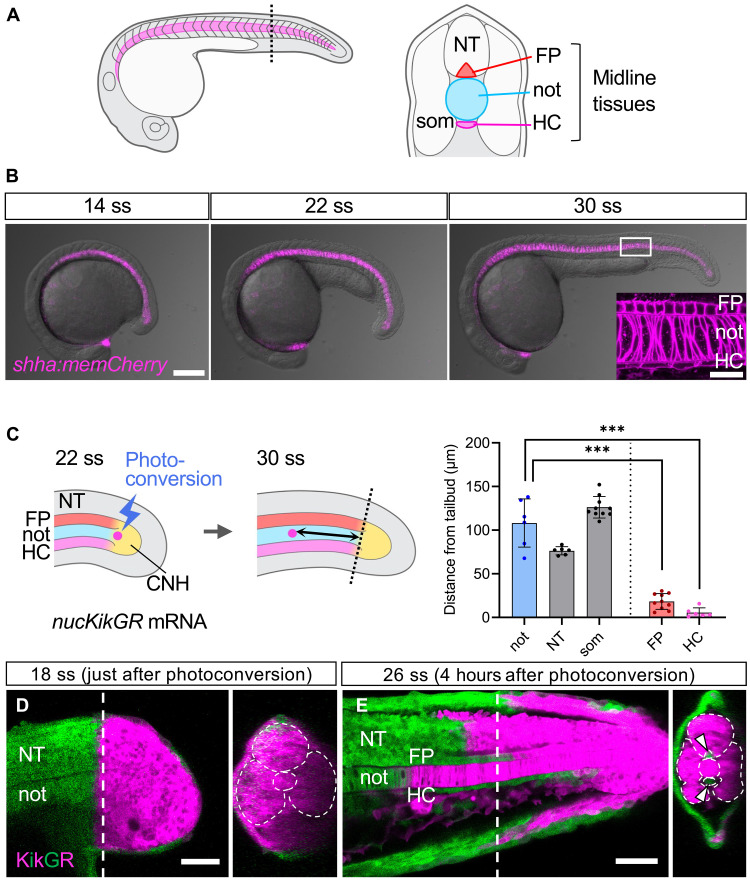
Midline tissues show distinct modes of elongation. (**A**) Left: Schematic of a 30–somite stage (ss) zebrafish embryo. Midline tissues are highlighted in magenta. Right: Schematic of a transverse view of the zebrafish trunk indicated by the dashed line in the left panel. (**B**) Lateral view of a zebrafish *shha:memCherry* embryo at 14, 22, and 30 ss. Inset in the right panel indicates a magnified confocal view of the region delineated by the white square, showing alignment of the midline tissues. (**C**) Left: Schematic of photoconversion of single cells in the tailbud expressing nuclear KikGR and memCherry. Dashed lines indicate the positions of the cells when they are photoconverted. Right: Distance from the tailbud after photoconversion in notochord (*n* = 6 embryos), neural tube (*n* = 6 embryos), presomitic mesoderm (*n* = 10 embryos), FP (*n* = 10 embryos), and HC (*n* = 6 embryos). (**D** and **E**) Photoconversion of cells in the tail region of an embryo expressing cytosolic KikGR. Dashed lines indicate the positions of optical sections shown on the right. Dashed closed lines delineate the neural tube, notochord, and somites. FP and HC cells have shifted relatively posteriorly to the rest of the neural tube and notochord at 26 ss (E). FP, floorplate; HC, hypochord; not, notochord; NT, neural tube; som, somites; CNH, chordoneural hinge. ***P < 0.001. Scale bars, 200 μm (B), 20 μm [inset in (B)], and 50 μm [(D) and (E)].

In developing vertebrate embryos, the notochord is a key structural element located in the middle of the body and generates a major biomechanical force driving body axis elongation (*14*–*18*). Dorsal to the notochord is the floorplate (FP) of the neural tube, and in anamniotes, the hypochord (HC) is located ventrally; these three tissues are collectively referred to as midline tissues (Fig. 1A) (*19*, *20*) and play fundamental roles in patterning the trunk structures via the secretion of morphogens (*21*–*25*). In zebrafish, the midline tissues undergo rapid posterior extension with precise length alignment to each other during the somitogenesis stage to form the main body axis (*14*, *19*), representing an excellent model for investigating the control system behind coordinated growth. Here, we investigate the elongation dynamics of zebrafish midline tissues and aim to identify a design principle for the coordinated growth of tissues within an embryo.

## RESULTS

### FP and HC show a distinct mode of elongation

In zebrafish, the notochord elongates rapidly within 8 hours at the somitogenesis stage, accompanied by the synchronous elongation of the FP and HC (Fig. 1B). Although notochord elongation has been attributed to the addition of cells from a progenitor pool called the chordoneural hinge (CNH) in the tailbud (*26*, *27*), the elongation dynamics of FP and HC has yet to be investigated. We first tracked cells in the posterior end of the trunk using time-lapse confocal microscopy. During the analysis, the viewpoint was fixed to the posteriorly moving CNH. Consistent with previous studies (*14*, *28*–*30*), we observed that notochordal and neural progenitor cells exited the tailbud region and gradually moved away from the CNH (fig. S1, A and B, blue and green cells) to contribute to the elongation of their respective tissues. However, we noted that the FP and HC cells remained adjacent to the CNH, following its posterior progression (fig. S1, A and B, red and magenta cells, and movie S1). When we quantified the displacement of each cell relative to the CNH using a photoconversion assay (Fig. 1C, left), the photoconverted notochordal cells as well as neural and somitic progenitor cells were located 108 ± 28 μm, 76 ± 5 μm, and 126 ± 12 μm (mean ± SD) away from the CNH, respectively, in the anterior direction after 4 hours (Fig. 1C, right, and fig. S1, C to H). In contrast, the photoconverted FP and HC cells shifted only 18 ± 9 μm and 6 ± 5 μm, respectively, indicating that these cells mostly stayed next to the CNH (Fig. 1C, right, and fig. S1, I to L). Thus, the FP, HC, and the CNH together create a stable tripartite formation in the tailbud. The differential displacement during body axis elongation resulted in shearing between tissues along the anterior-posterior axis, as manifested by the misalignment of photoconverted regions within the FP and neural progenitor cells that express KikGR (Fig. 1, D and E). Together, these results show that the FP and HC exhibit a distinct mode of tissue elongation compared to other trunk tissues; they do not elongate by accretion of cells from the CNH, but instead appeared to follow the posterior movement of the CNH.

### FP and HC cells exhibit migratory behavior

**Fig. 2.**
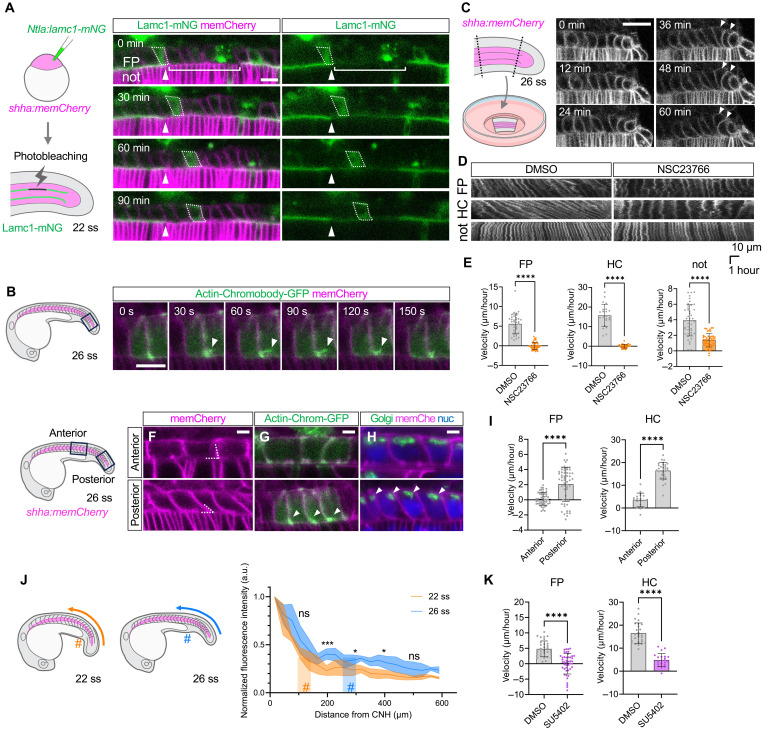
FP and HC cells collectively migrate posteriorly on the notochord. (**A**) Left: Schematic of photobleaching after injection of laminin c1-mNeonGreen plasmid into a *shha:memCherry* embryo. Right: Time-lapse images of FP cells after photobleaching (brackets). A representative FP cell is outlined (dashed lines). The anterior edge of the photobleached region is marked (arrowheads). (**B**) Time-lapse images of an FP cell expressing Actin-Chromobody-GFP, showing a dynamic protrusion (arrowheads). (**C**) Left: Schematic of explant preparation from the *shha:memCherry* tailbud. Right: Time-lapse images showing posterior FP cell squeezing (arrowheads). (**D**) Kymographs of FP, HC, and notochord cells in explants treated with DMSO or NSC23766. (**E**) Velocity of FP, HC, and notochord cells in DMSO (FP: *n* = 4 embryos, *N* = 37 cells; HC: *n* = 4 embryos, *N* = 22 cells) or NSC23766 (FP: *n* = 4 embryos, *N* = 40 cells; HC: *n* = 4 embryos, *N* = 31 cells). (**F** to **H**) Anterior and posterior FP cells showing cell contours [(F), dotted lines], cytoplasmic actin [(G), arrowheads], and Golgi apparatus [(H), arrowheads]. (**I**) Velocity of FP and HC cells in anterior and posterior explants. (**J**) Left: Schematics of 22-ss and 26-ss embryos showing the measurement axis for *etv4* expression. “#” indicates the position of the cloaca. Right: Normalized *etv4* HCR signal profiles along the anterior-posterior axis from the CNH at 22 ss (orange, *n* = 4 embryos) and 26 ss (blue, *n* = 4 embryos), measured along the anterior-posterior axis from the CNH. Statistical differences at 100 to 500 μm (two-tailed Welch’s *t* test). a.u., arbitrary units. (**K**) Velocity of FP and HC cells in DMSO or SU5402. FP, floorplate; HC, hypochord; not, notochord; BM, basement membrane. ns, not significant; *P < 0.05; ***P < 0.001; ****P < 0.0001. Scale bars, 10 μm [(A) and (B)], 20 μm (C), and 5 μm [(F) to (H)].

The above findings prompted us to investigate how the FP and HC could follow the CNH. During time-lapse imaging of the tailbud, we noticed that multiple FP cells appeared to crawl on the notochord in the posterior direction (movie S2). To examine whether the FP and HC cells migrate on the notochord, we partially labeled the extracellular matrix (ECM) component laminin, located between the FP or HC and the notochord, by photobleaching (Fig. 2A, left). Time-lapse imaging showed that the FP and HC cells gradually shifted toward the posterior side relative to photobleached laminin as axis elongation proceeded (Fig. 2A, right, and fig. S2A), indicating the migratory activity of these cells. Consistent with this, when we examined the actin distribution within the FP and HC cells, we found that the cells tend to accumulate actin on the posterior basal side (Fig. 2B and fig. S2B), with temporary formation of filamentous actin-rich protrusions lasting a few minutes (Fig. 2B, arrowheads, and movie S3). These biased protrusions on the posterior-basal side were corroborated by electron microscopy (fig. S2, C to F).

Furthermore, to investigate the autonomy of their migratory activity, we surgically isolated tail fragments containing the FP and HC cells without the CNH region and cultured them ex vivo (Fig. 2C, left). This revealed that the FP and HC cells collectively migrated posteriorly, as indicated by the kymographs (fig. S3, A and B). The FP and HC cells at the posterior end of the explant eventually became packed and squeezed (Fig. 2C, right, and movie S4), likely due to mechanical compression from migrating anterior cells, while such squeezing was never observed at the anterior end. The migration activity observed ex vivo indicates that the posterior movement is not caused by a pushing force from the anterior (rostral) region or a dragging force from the posteriorly moving CNH.

As cell migration often involves Rac1-mediated actin reorganization (*31*), we tested whether a Rac1-specific inhibitor, NSC23766, disrupts their migratory behavior. In the presence of NSC23766, the velocity of FP and HC cells nearly halted (Fig. 2, D and E). Because NSC23766 affects the entire explant, it also mildly affected the notochord (Fig. 2, D and E). However, in our explant setup, FP and HC were separated from the notochord by a laminin-rich ECM, minimizing direct physical interactions. Therefore, we conclude that NSC23766 primarily acted directly on FP and HC cells, supporting the idea that FP and HC actively migrate posteriorly via Rac1.

We next asked whether there is any spatial difference in the migratory activity of the FP and HC cells along the anterior-posterior axis. We found that the migratory activity exhibits a posterior-to-anterior gradient by several criteria. First, the posterior cells were more oblique with sharper angles between the lateral edges and the basal surface (Fig. 2F and fig. S3C). Second, the enrichment of actin at the posterior-basal corner was more pronounced in the posterior cells (Fig. 2G and fig. S3D). Third, the Golgi apparatus, whose position often correlates with the direction of active cell migration (*32*–*35*), was also polarized to the posterior side of the posterior cells, while it was more evenly distributed in the anterior cells (Fig. 2H and fig. S3E). Last, posterior explants showed more motile FP and HC cells (Fig. 2I).

Previous studies have reported that a spatial gradient of fibroblast growth factor (FGF) signaling, which is high in the tailbud and decreases anteriorly, is essential for the posterior extension of the paraxial mesoderm and contractile movement of the endoderm (*36*, *37*). We postulated that FGF signaling regulates the graded migratory activity in FP and HC. *dusp6* and *etv4*, downstream genes involved in FGF signaling, exhibited an expression gradient within FP and HC (fig. S4, A, B, and D to H), with its effective length expanded as the tissue elongated (Fig. 2J). When we disrupted FGF signaling with SU5402 treatment, the FP and HC cells in the posterior explants halted their migration in the posterior direction and some FP cells apparently started to be dragged anteriorly on the ECM connecting to the vacuolating notochord cells (Fig. 2K), indicating the involvement of FGF signaling in their migratory behavior. Furthermore, the expression of *fgf8a* was strong in the CNH including the posterior terminus of the notochord (fig. S4C), implying the involvement of the CNH in providing graded migration activity in FP and HC. We thus conclude that FP and HC cells actively migrate posteriorly in a graded manner in response to FGF signaling, likely under the control of the CNH.

### Collective cell migration in FP and HC leads to Yap-mediated cell proliferation

How does active migration of FP and HC cells lead to tissue elongation and ultimately coordinated growth with the notochord? Because previous studies have indicated that migratory cells in vitro can stretch neighboring cells and exert mechanical tension (*35*, *38*), we hypothesized that collective migration causes cellular tension within the FP and HC, resulting in cell proliferation. Time-lapse imaging revealed that FP and HC cells frequently divide, with their daughter cells being reintegrated into the tissues (fig. S5A and movie S5). Accordingly, 5-ethynyl-2′-deoxyuridine (EdU) labeling mosaically marked the nuclei of FP and HC cells, from the posterior side near the tailbud to the anterior at the level of yolk extension, indicating a broad distribution of cell proliferation along the entire anterior-posterior axis of the posterior trunk (fig. S5B, C).

**Fig. 3.**
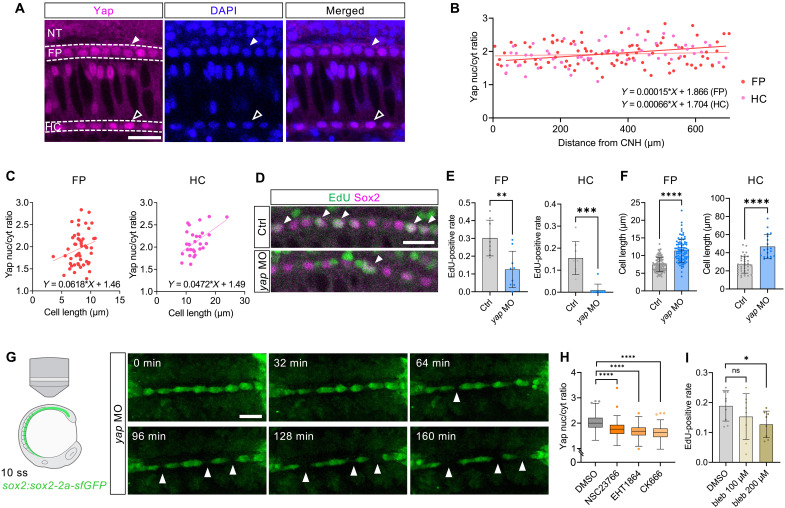
Yap mediates cell proliferation in FP and HC during tissue elongation. (**A**) Yap protein distribution at 26 ss. Nuclear localization in FP and HC cells (dashed lines) is indicated by filled and open arrowheads, respectively. (**B**) Scatter plot showing the Yap nuclear-to-cytoplasmic ratio in FP (red, *n* = 97 cells) and HC (magenta, *n* = 58 cells) cells along the anterior-posterior axis with corresponding regression lines. (**C**) Scatter plots showing cell length versus Yap nuclear-to-cytoplasmic ratio in FP (*n* = 50 cells) and HC (*n* = 28 cells). Pearson’s correlation coefficient *r* = 0.232 (H, *P* = 0.106) and 0.514 (I, *P* = 0.005). (**D**) EdU staining of FP cells of 26-ss embryos injected with control or *yap* morpholino oligos. Arrowheads indicate EdU-positive cells. (**E**) EdU-positive fractions of FP and HC cells in control (*n* = 8 embryos) and *yap* morphants (*n* = 9 embryos). (**F**) Lengths of FP and HC cells in control (*N* = 4 embryos; *n* = 111 cells for FP, 36 cells for HC) and *yap* morphants (*N* = 4 embryos; *n* = 89 cells for FP, 19 cells for HC). (**G**) Dorsal view of a *sox2:sox2-2a-sfGFP yap* morphant embryo showing HC cells. Arrowheads indicate cell gaps. (**H**) Yap nuclear-to-cytoplasmic ratio of FP cells when treated with DMSO (*N* = 7 embryos, *n* = 182 cells), NSC23766 (*N* = 8 embryos, *n* = 210 cells), EHT1864 (*N* = 6 embryos, *n* = 159 cells), or CK666 (*N* = 7 embryos, *n* = 167 cells). (**I**) EdU-positive fractions of FP cells in the DMSO-treated (*n* = 9 embryos) and blebbistatin-treated (*n* = 8) embryos. Anterior to the left. FP, floorplate; HC, hypochord; NT, neural tube. ns, not significant; *P < 0.05, **P < 0.01, ***P < 0.001, ****P < 0.0001. Scale bars, 20 μm [(A) and (D)] and 50 μm (G).

Yap is a well-known transcriptional cofactor that regulates cell proliferation via mechanical tension in various cell types (*4*, *39*–*42*) by activating downstream genes upon its translocation to the nucleus. We found that Yap protein is enriched in the nuclei of FP and HC cells (Fig. 3A). The Yap reporter line *Tg(4xGTIIC:EGFP)* (*43*) also displayed fluorescence in these midline tissues (fig. S5D). Yap nuclear localization was mostly uniform along the anterior-posterior axis of FP and HC (Fig. 3B), indicating no substantial positional bias in these tissues. However, when analyzed as a function of cell size, nuclear Yap levels positively correlated with cell length in FP and HC (Fig. 3C), suggesting that mechanical strain on individual cells enhances Yap activity. Knockdown of *yap* decreased the fraction of cells incorporating EdU in the FP and HC (Fig. 3, D and E) and increased cell length along the anterior-posterior axis (Fig. 3F and fig. S5E) without affecting their cell fates (fig. S6). Notably, visualization of cellular behavior revealed that the continuity of the HC cells was eventually disrupted in *yap* morphants, resulting in gaps between the cells (Fig. 3G and fig. S5F), suggesting that Yap-mediated cell proliferation is essential for their continuous growth. Treatment with NSC23766, or other migration inhibitors such as another Rac1 blocker EHT1864 or an Arp2/3 inhibitor CK666, in whole embryos reduced the translocation of Yap protein to the nucleus (Fig. 3H), indicating that posterior migration enhances Yap activity. Furthermore, perturbing cellular tension with blebbistatin, a specific inhibitor of myosin II mediating cell contractility, resulted in a concentration-dependent decrease in the frequency of EdU-positive cells (Fig. 3I). Collectively, these data suggest that collective cell migration within the FP and HC mediates cell stretching and cell proliferation, leading to elongation of the tissues.

To evaluate the role of graded migratory activity in a quantitative manner, we built a 2D vertex model depicting the elongation dynamics of linearly aligned cells representing the FP or HC. We assumed that cell division occurs when the elastic energy of a cell exceeds a threshold in response to tensile forces from neighboring cells (fig. S7A; see Materials and Methods and Supplementary Text). When the cells were set to be nonmigratory except for the posterior tip, cell proliferation was restricted to the posterior-most region while other cells were nonproliferative (fig. S7B and movie S6), consistent with a previous simulation study (*44*). When migratory activity was provided to all cells at the same velocity, the anterior-most region experienced excessive tensile force that could not be compensated by cell divisions, resulting in gaps at the anterior end (fig. S7C; movie S7), similar to the previous model (*44*). In contrast, when the migration activity of the cells was graded, mechanical forces were broadly distributed among cells along the entire anterior-posterior axis, and spatially unbiased cell proliferation was achieved (fig. S7, D and E, and movie S8), in agreement with the EdU staining result (fig. S5C). Consequently, graded migration yielded rapid tissue growth compared to the nongraded mode. These results suggest that collective cell migration with graded activity allows for cell proliferation broadly distributed along the anterior-posterior axis, thus enabling rapid elongation of the FP and HC. Our observation that the *etv4* expression gradient extends as the tissue expands (see Fig. 2J) implies that this system scales in concert with body axis elongation. Together, this elongation mechanism driven by collective cell migration provides the mechanistic basis for the FP and HC to catch up with the growing notochord, ensuring establishment and maintenance of the tripartite formation of the midline tissues.

### Cadherin-mediated multitissue formation at the CNH facilitates fine-tuning of coordinated growth

**Fig. 4.**
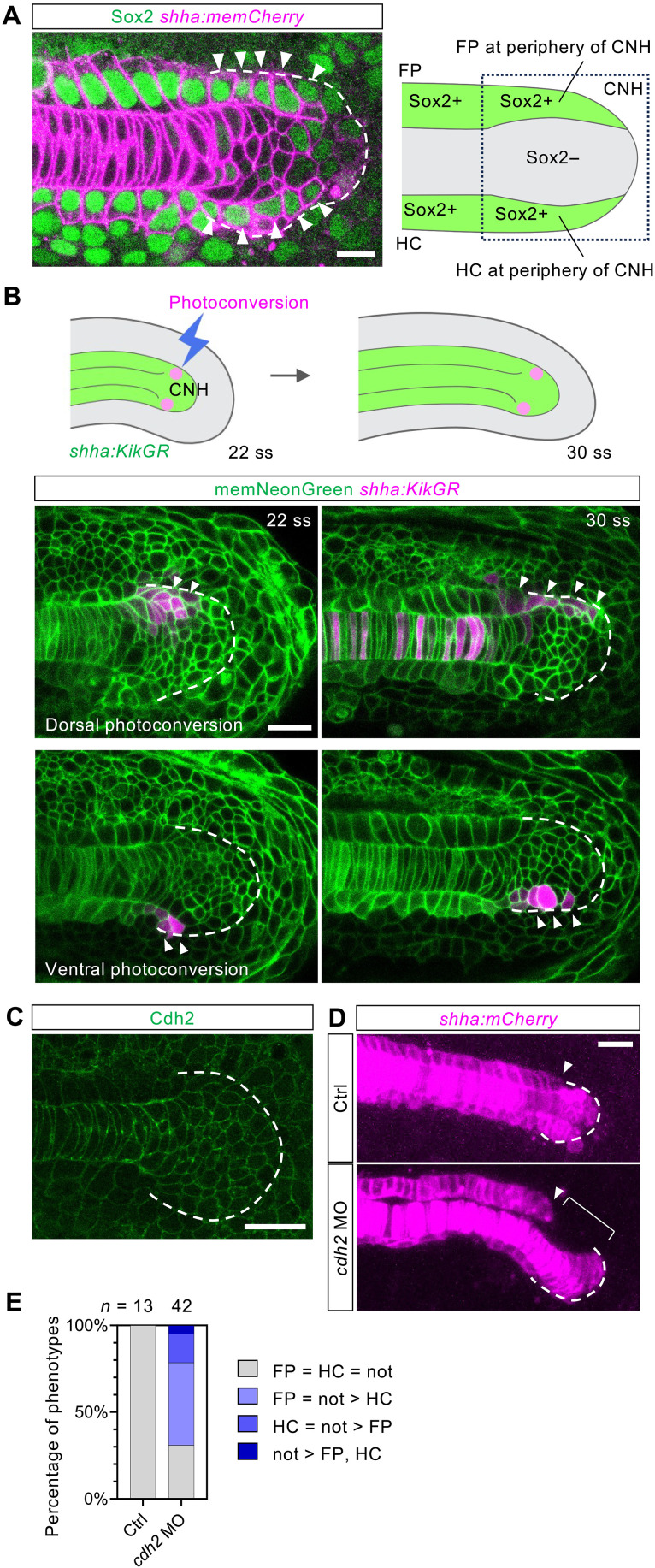
FP and HC are tethered to the notochord at the CNH. (**A**) Left: Distribution of Sox2-positive cells in the tailbud of a 30-ss *shha:memCherry* embryo. Cells with distinct Sox2 signals at the periphery of the CNH are indicated by arrowheads. The dashed line delineates the CNH. Right: Schematic of the distribution of cells in the CNH. (**B**) Top: Schematic of the photoconversion experiment using *shha:KikGR* embryos. Bottom: Photoconverted cells (arrowheads) in the CNH (outlined with dashed lines) at 22 ss (just after photoconversion) and 30 ss (4 hours later). (**C**) Cdh2 protein distribution around the CNH. (**D**) Morphology of the midline tissues of *shha:memCherry* embryos injected with control (left) or *cdh2* (right) morpholino oligos. Arrowheads indicate the posterior terminus of the FP. The bracket indicates a gap between the FP and the notochord. Dashed lines delineate the CNH. (**E**) Quantification of defects in unmatching the midline tissues in control or *cdh2* morphants. Scale bars, 10 μm (A) and 20 μm [(B) to (D)].

Our analyses on cell behaviors in the tailbud indicated that the FP and HC build a stable tripartite formation together with the extending notochord at the CNH. To investigate how the migrating FP and HC cells can establish a robust formation with the notochord cells, we focused on the structural organization of the midline tissues around the CNH at the cellular level. By visualizing how FP and HC cells are in contact with the notochord cells using immunohistochemistry, we found that the CNH cell population, delineated by the fluorescent signal of *shha:memCherry* (membrane-localized mCherry), contained these tissues in a nested manner; the CNH consists of Sox2-negative cells representing notochord progenitor cells in the center and Sox2-positive cells surrounding this notochord progenitor group (Fig. 4A, arrowheads, and fig. S8, A and B). When we labeled the Sox2-positive cells in the CNH by photoconversion using a *shha:KikGR* transgenic line (Fig. 4B, top), the labeled cells mostly remained at the periphery of the CNH (Fig. 4B, bottom), suggesting that these cells stably attach to the notochord progenitor cells throughout the elongation process.

The interface between the surrounding cells and the notochord progenitor cells was devoid of major ECM components, laminin and fibronectin (fig. S8, C and D), implying their direct contact. Instead, we found that a cell-cell adhesion molecule, cadherin 2 (Cdh2), is highly expressed in the entire region of the CNH (Fig. 4C). Knockdown of *cdh2* disrupted cell adhesion between the FP and CNH and between the HC and CNH, which resulted in the detachment of FP and HC cells from the notochord (Fig. 4D). Subsequently, these *cdh2*-deficient embryos often exhibited unmatched lengths of the midline tissues, with FP and HC being shorter than the notochord (Fig. 4E). Together, these data show that FP and HC are directly in contact with the notochord progenitor cells at the CNH and adhered by Cdh2 to establish a stable intertissue formation between FP, HC, and the notochord.

**Fig. 5.**
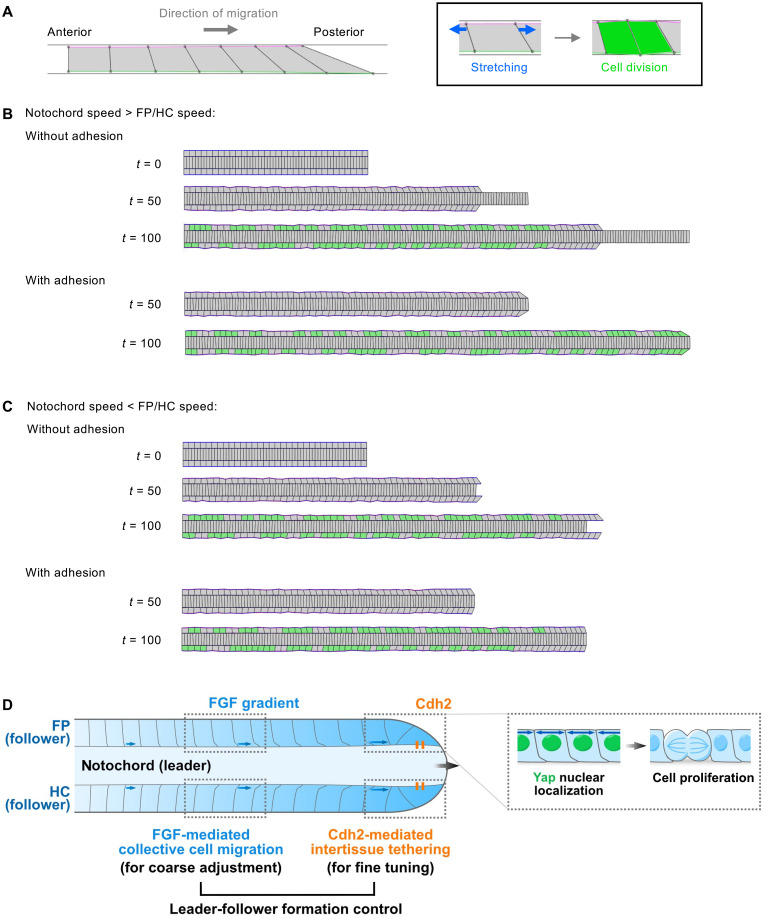
Assembly of a multitissue formation facilitates coordinated elongation of the midline tissues. (**A**) Schematic of mechanical stretch-mediated cell division in the computational model. (**B** and **C**) Simulated elongation of the three midline tissues with (top) or without (bottom) intertissue adhesion to create a formation of the posterior ends. Two cases where the elongation speed of the notochord is faster (B) or slower (C) than the elongation speeds of FP and HC are shown. Cells that experienced mitoses from *t* = 50 onward are colored green. (**D**) Mechanistic model for coordinated elongation of the midline tissues driven by leader-follower formation control. See the Results for details.

Last, we asked how this intertissue formation could control the lengths of the FP and HC with the notochord during growth. To this end, we created a vertex model whereby collectively migratory FP/HC tissues are connected to a growing tissue characterizing the notochord at the posterior-most side (Fig. 5A). With this model, we set the FP/HC to collectively migrate so that they grow at a faster or slower pace compared to the notochord. As expected, without assuming intertissue adhesion to make a formation, FP and HC elongated independently of the notochord, resulting in unmatched lengths between the midline tissues (Fig. 5, B and C, top, and movies S9 and S10). However, in the presence of physical association between the posterior termini of the tissues to create a formation, the migrating FP/HC tissues elongated in accordance with the notochordal tissue regardless of the relative speed of notochordal growth (Fig. 5, B and C, bottom, and movies S11 and S12). Cell proliferation in the FP/HC was attenuated or enhanced locally at the posterior termini when they migrated faster or more slowly than the notochord, respectively, indicating that their synchronous growth is achieved via local modification of cell division frequencies and subsequent release of mechanical tensions, or mechanical buffering. In conclusion, our data demonstrate that collective cell migration in FP and HC, with their posterior ends tethered to the notochord, achieves coordinated elongation with the notochord. This system allows for maintaining a formation of cells across the three tissues throughout body axis elongation.

## DISCUSSION

During embryonic development, the growth of multiple tissues must be coordinated to generate structurally integrated assemblies with desired functions. Design principles for orchestrating the growth of multitissue assemblies are currently lacking. We suggest that formation control theory represents a framework for understanding how different types of cells can interact as a system to create complex patterns and shapes in a precise and robust fashion. Formation control systems differ in terms of how much interagent communication is required. Example control schemes include “leader-follower,” in which followers track the leader; “behavioral,” in which agents maintain behaviors such as cohesion and collision avoidance; and “structural,” in which the movement of the overall formation is given (*45*, *46*).

In our system, both FGF-mediated collective migration and Cdh2-mediated intertissue tethering fit the leader-follower model, where FP and HC cells follow the notochord as the leading structure (Fig. 5D). In this model, the notochord acts as the leader and autonomously extends through addition of cells at its posterior end. The FP and HC serve as followers relying on multiple cues from the leader. First, they migrate along the nascent notochord ECM in a graded fashion in response to FGF from the CNH at the extending tip of the notochord. This graded migration propagates mechanical tension throughout the FP and HC cells themselves, which, in turn, promotes Yap-mediated proliferation. Second, Cdh2 expression at the CNH tethers FP and HC to the notochord, enabling local mechanical coupling at the posterior ends to likely modulate the speed of collective cell migration. Together, these two processes synergistically work to achieve the coordinated growth of the tissues. Our data suggest that during axis elongation, the region where these mechanisms operate extends posteriorly along with tissue growth. In the presomitic mesoderm, scaling of the FGF signaling gradient has been reported (*47*), where antagonistic molecules counteracting the FGF source contribute to this scaling process (*48*). A similar scaling mechanism may operate in the midline tissues, potentially allowing FGF signaling to adjust dynamically as the posterior trunk elongates.

Long-range formation control via FGF signaling provides coarse adjustment of the relative positions of the midline tissues, while short-range, Cdh2-mediated formation control offers fine-tuning of these positions at the posterior end. We speculate that similar formation control mechanisms may be broadly used in growing organisms to coordinate multitissue growth, as seen recently in the heart for myocardium and endocardium (*4*).

## MATERIALS AND METHODS

### Fish husbandry

The zebrafish RW, AB, and TL strains were used as wild types. The transgenic lines *Tg(actb2:mem-mCherry2)*^hm29^ (*49*), *Tg(4xGTIIC:EGFP)*^mw61^ (*43*), and *Tg(sox2:sox2-2a-sfGFP)*^stl84^ (*50*) were also used. Adult fish were kept at 28°C under a 14-hour light/10-hour dark cycle. Fertilized embryos were incubated in 1/3× Ringer’s solution (38.7 mM NaCl, 0.97 mM KCl, 1.67 mM Hepes, and 1.80 mM CaCl_2_) at 23° to 28°C to achieve the desired developmental stages (*51*). All fish husbandry and experimental procedures were performed under approvals from the Harvard Medical School Animal Care and Use Committee (Protocol #04487), the University of Tokyo (Protocol #20-2), and the Institute of Science Tokyo (Protocol #I2020010).

### Plasmid construction

All plasmid construction was performed using isothermal assembly of PCR-amplified DNA fragments with NEBuilder (New England Biolabs) or a mixture of T5 exonuclease (T5E4111K, Epicentre), Taq DNA ligase (New England Biolabs), and Phusion DNA polymerase (New England Biolabs) [for details, see Gibson *et al.* (*52*)]. The plasmid pMT-*2.4shha-ABC:memCherry* was constructed by combining the promoter and enhancer sequences of the *shha* gene from p*2.2shh:gfp:ABC* (*53*), the mCherry coding sequence, two copies of the membrane localization signal, and the backbone from pMTB-*memCherry* (*49*) using isothermal assembly. The pMT-*2.4shha-ABC:KikGR* construct was created by replacing the memCherry sequence in pMT-*2.4shha-ABC:memCherry* with KikGR amplified from pTOPO-NLS-KikGR (gift from D. Ohtsuka). For pCS2-*KikGR* and pCS2-*nuc-KikGR*, the KikGR or NLS-KikGR sequence was inserted into the pCS2+ backbone. For pMTB-*Actin-Chromobody-GFP*, the coding sequence of pMTB-*memCherry* was replaced with Actin-Chromobody-TagGFP2 of the Actin-Chromobody plasmid (ChromoTek). For p*Ntla:lamc1-mNeonGreen*, the coding region of the zebrafish *lamc1* gene was cloned from a zebrafish cDNA pool with the primers (forward: ATGTCGCTTTTTAGCTGTTTGTTGTTATGG, reverse: TGGCCGCTCTAGAGACGGC) and tagged at its C terminus with the mNeonGreen sequence amplified from pMTB-*mem-mNeonGreen-mNeonGreen* (*54*) placed downstream of the 1-kb *ntla* promoter derived from *ntl:CFP* (gift from S. Harvey) (*55*) and integrated with the pMT backbone from pMTB-*memCherry*.

### Transgenesis

Transgenic lines *Tg(shha:memCherry)* and *Tg(shha:mKikGR)* were generated by injecting plasmids pMT-*2.4shha-ABC:memCherry* and pMT-*2.4shha-ABC:mKikGR*, respectively, along with Tol2 transposase mRNA synthesized from pCS-TP (*56*) using the mMESSAGE mMACHINE SP6 Transcription Kit (Thermo Fisher Scientific) into wild-type embryos at the one-cell stage. Fluorescent embryos were selected and raised. Upon reaching sexual maturity, these fish were outcrossed with wild-type fish and screened for founders. Founders were isolated and raised to establish single allele lines.

### mRNA, plasmid, and morpholino injection

mRNAs were synthesized from pCS2-*KikGR*, pCS2-*nuc-KikGR*, pMTB-*Actin-Chromobody-GFP*, *mNeonGreen-Giantin* (gift from D. Gadella, Addgene #98880) (*57*), and pMTB-T7-α*-bungarotoxin* (*58*) plasmids using the mMESSAGE mMACHINE SP6 or T7 Transcription Kit (Thermo Fisher Scientific) after linearization. mRNA was injected at concentrations of 30 to 100 ng/μl, mixed with phenol red solution (P0290, Sigma-Aldrich), into one-cell stage embryos (eight-cell stage for Actin-Chromobody-GFP to achieve mosaic expression). *bungarotoxin* mRNA was coinjected to immobilize embryos during time-lapse imaging. Fluorescent embryos were screened under a fluorescent stereomicroscope the following day before imaging.

For plasmid injection, one-cell stage embryos were injected with p*Ntla:lamc1-mNeonGreen* (25 ng/μl) and *Tol2* mRNA (25 ng/μl) and screened for bright fluorescence the next day. For morpholino antisense oligonucleotide (MO) injection, previously characterized MOs targeting *yap1* (target sequence: AGCAACATTAACAACTCACTTTAGG) (*59*) and *cdh2* (target sequence: TCTGTATAAAGAAACCGATAGAGTT) (*60*) were obtained from GeneTools along with the standard control MO. *yap1* and *cdh2* MOs were injected at concentrations of 1 mM and 250 μM, respectively, mixed with phenol red solution into one-cell stage embryos. All injection experiments were performed using Nanoject II (Drummond).

### Image acquisition

For live imaging, dechorionated embryos were mounted on the lateral side in a 1% agarose gel chamber shaped by a custom-made plastic lateral mount obtained from Shapeways [for details, see Megason (*61*)], filled with 1/3× Ringer’s solution in a 35-mm plastic or glass-based dish (P60G-1.5-30-F, Phoenix Science). Embryos were rotated for proper positioning with hair loops before gently lowering a coverslip. Care was taken to ensure that embryos were not compressed by the coverslip or constrained by the chamber, enabling imaging of their normal proportions. Live imaging was performed using an LSM710 confocal microscope (Carl Zeiss) equipped with 40×, 25×, or 20× water-immersion objective lenses (LD C-Apochromat 40×/1.1 W Korr M27; LD LCI Plan-Apochromat 25×/0.8 Imm Corr DIC M27; W Plan-Apochromat 20×/1.0 DIC M27, Carl Zeiss). Time-lapse imaging was conducted either in a home-built incubator or in a stage-top incubator (INUCP-Kri, Tokai Hit) at 28.5°C with 3- or 4-min time intervals. For imaging fixed embryos, embryos were either mounted in the 1% agarose gel chamber or embedded in 1% low–melting point agarose (Sigma-Aldrich) in phosphate-buffered saline (PBS) in a 35-mm glass-based dish and observed under the confocal microscope. Movies were generated using Draw Arrow in Movies plugin (*62*) in Fiji (*63*).

### Photoconversion and photobleaching

To photoconvert a specific region of embryos injected with *KikGR* or *nuc-KikGR* mRNA or *shha:KikGR* transgenic embryos, the embryos were mounted laterally in a 1% agarose gel chamber under the LSM710 confocal microscope. The region to be photoconverted was defined by drawing a polygon using the Region function in the Zen software (Carl Zeiss) and was then exposed to a 405-nm laser at approximately 2% power for 10 to 30 s. To photobleach a specific region of Lamc1-mNeonGreen signals, the region was similarly defined using the Region function and then exposed to a 488-nm laser at approximately 50% power for 30 s.

### Whole-mount in situ hybridization

Whole-mount in situ hybridization was performed as described previously (*64*) with minor modifications. Embryos were fixed in 4% paraformaldehyde (PFA) for 1 hour at room temperature, followed by overnight fixation at 4°C. After dehydration in 100% methanol for 15 min at room temperature, embryos were stored at −20°C. Rehydration was performed through a graded methanol/PBST (0.1% Tween 20 in PBS) series: 75, 50, and 25% methanol/PBST for 5 min each, followed by two 5-min washes in PBST. Embryos were permeabilized with proteinase K (10 μg/ml) in PBST for 10 min at room temperature, then postfixed in 4% PFA for 20 min, followed by four 5-min PBST washes. Prehybridization was conducted in hybridization buffer [50% formamide, 5× saline sodium citrate buffer (SSC), torula tRNA (500 μg/ml), heparin (50 μg/ml), and 0.1% Tween 20, pH 6.5] at 60°C for 1 hour. Denatured probes (100 ng) were hybridized overnight at 60°C in hybridization buffer containing 5% dextran sulfate (D6001, Sigma-Aldrich). Embryos were then washed at 60°C twice with 50% formamide/2× SSC/0.1% Tween 20 for 30 min, once with 2× SSC/0.1% Tween 20 for 15 min, and twice with 0.2× SSC/0.1% Tween 20 for 30 min. After rinsing in MABT [0.1% Tween 20 in maleic buffer (MAB; 100 mM maleic acid and 150 mM NaCl, pH 7.5)], embryos were blocked in 2% blocking reagent (11096176001, Roche) in MAB/0.1% Tween 20 for 1 hour, then incubated overnight at 4°C with anti-DIG-AP antibody (1/7000 dilution). Following five 15-min washes in MABT and two 5-min washes in NTMT (1 M tris-HCl, pH 9.5, 5 M NaCl, 1 M MgCl_2_, and 0.1% Tween 20), staining was developed in nitro blue tetrazolium (NBT)/5-bromo-4-chloro-3-indolyl-phosphate (BCIP) solution [NBT (0.34 mg/ml) and BCIP (0.18 mg/ml) in NTMT] and stopped with 4% PFA/PBS.

RNA probes were synthesized using the following primers: *col2a1a* (5′-GGATCTGATGGTCCACCTGG-3′ and 5′-GCTTGGTCCATGTAAGCCAC-3′), *foxa1* (5′-ACTTTCAGGAGGAGTTACCCG-3′ and 5′-GAGAACAGGCCTGGAATACAC-3′), *ntn1b* (5′-TTTTGGTAACGTGCGTCTCC-3′ and 5′-TTAACGGCTGCATGTCCG-3′), and *gdf6a* (5′-AGGATTCCCGAAAAGCAGAATG-3′ and 5′-ACAGTATACTGATGGGGCTGAG-3′).

### In situ hybridization chain reaction

Hybridization chain reaction (HCR) probes and buffers were obtained from Molecular Instruments, except for the *actb2* probe, which was designed using a custom program and synthesized by IDT. HCR analysis was performed following the standard protocol (*65*). Briefly, dechorionated embryos were fixed overnight at 4°C in 4% PFA in PBS, rinsed in PBS, and then transferred to methanol for storage at −20°C until further use. For rehydration, embryos underwent a graded series of methanol:PBST dilutions at concentrations of 75, 50, and 25, each for 5 min, followed by two 5-min washes in PBST. Embryos were then incubated in prewarmed probe hybridization buffer at 37°C for 30 min with rocking and hybridized with 2 pmol of probes in probe hybridization buffer at 37°C for at least 12 hours. The next day, embryos were washed in probe wash buffer at 37°C four times for 15 min each, followed by two 15-min washes in SSCT (15 mM sodium citrate, 150 mM NaCl, and 0.1% Tween 20) at room temperature. Subsequently, embryos were incubated with amplification buffer for 30 min at room temperature, and then with a set of fluorescently labeled h1 and h2 hairpin amplifiers at room temperature overnight, protected from light. This was followed by two 5-min washes, two 30-min washes, and one 5-min wash in 5× SSCT, and lastly two 5-min washes in PBST at room temperature.

For subsequent immunohistochemistry to detect mCherry signals, embryos were incubated in blocking solution (2% BSA in PBS with 0.1% Tween 20) for 30 min and then in blocking solution containing anti-mCherry antibody (1/1000, LS-C204207, LifeSpan Biosciences) overnight at 4°C. After four rounds of 30-min washes in PBST, embryos were incubated in blocking solution with anti-goat IgG conjugated with Alexa Fluor 555 (1/1000, A21432, Thermo Fisher Scientific).

### Immunohistochemistry

Dechorionated embryos at desired stages were fixed overnight at 4°C in 4% PFA in PBS, rinsed, and then stored in PBS at 4°C until further use. Embryos were permeabilized with 1% Triton X-100 in PBS for 1 hour (2 hours for staining Yap), blocked in 2% BSA in PBSDT [PBS with 1% dimethyl sulfoxide (DMSO) and 0.1% Triton X-100] for 1 hour (2 hours for staining Yap), and then incubated overnight at 4°C with the blocking solution containing primary antibodies against Sox2 (1/200, ab92494, Abcam; 1/200, GTX124477, GeneTex), mCherry (1/1000, LS-C204207, LifeSpan Biosciences), Yap (1/200, 13584-1-AP, Proteintech), or Cdh2 (1/200, GTX125885, GeneTex). Following this, embryos were washed in PBSDT five times for 15 min each and incubated overnight at 4°C with the following secondary antibodies: anti-rabbit IgG conjugated with Alexa Fluor 405, 488, or 568 (1/500, A31556, A21206, A21441, or A10042, Thermo Fisher Scientific) and anti-goat IgG conjugated with Alexa Fluor 555 (1/500, A21432, Thermo Fisher Scientific). Counterstaining was performed with rhodamine-phalloidin (R415, Invitrogen) or 4′,6-diamidino-2-phenylindole in the secondary antibody solution. After washing four times for 15 min each in PBSDT, embryos were mounted in 1% low–melting point agarose in PBS on a glass-based dish and imaged as described above.

For laminin and fibronectin staining, embryos were initially fixed with 2% PFA in PBS for 2 hours at room temperature and then overnight at 4°C. After fixation, embryos were quenched with 50 mM glycine in PBSTT (PBS with 0.5% Triton X-100 and 0.5% Tween 20) and washed in PBSTT four times for 30 min each. Blocking was performed with 5% BSA in PBSTT, followed by incubation in 2% BSA in PBSTT with anti-fibronectin (1/100, F3648, Sigma-Aldrich) or anti-laminin (1/100, L9393, Sigma-Aldrich) antibodies. Embryos were washed four times for 30 min each in 2% BSA in PBSTT and then incubated with anti-rabbit IgG conjugated with Alexa Fluor 594 (1/500, A11012, Thermo Fisher Scientific) and Alexa Fluor 488 phalloidin (1/500, A12379, Thermo Fisher Scientific).

### EdU staining

To detect cell proliferation, we used the Click-iT EdU Cell Proliferation Kit for Imaging (C10637, Thermo Fisher Scientific) and followed the manufacturer’s instruction with the following modifications. Dechorionated embryos were prechilled on ice for 15 min in 1/3× Ringer’s solution. For EdU incorporation, embryos were incubated in ice-cold EdU solution for 30 min on ice with shaking. Occasional vigorous agitation (once every 3 to 5 min) was applied to ensure uniform staining across embryos. Afterward, embryos were transferred to 1/3× Ringer’s solution maintained at 28.5°C and incubated for an additional 5 min to enhance EdU incorporation. The embryos were then fixed in 4% PFA in PBS for 2 hours at room temperature, followed by two washes with PBS. For permeabilization, embryos were treated with 1% Triton X-100 in PBS for 1 hour. Staining was performed by incubating embryos in staining solution for 30 min in the dark to detect incorporated EdU. The staining reaction was stopped by washing the embryos twice with PBSDT. To perform immunohistochemistry subsequently, embryos were blocked in 2% BSA in PBSDT for 30 min at room temperature to prevent nonspecific antibody binding, followed by incubation with primary antibodies as described in the “Immunohistochemistry” section.

### Transmission electron microscopy

Dechorionated RW embryos at 26 somite stage (ss) were fixed overnight at 4°C with 2% PFA and 2% glutaraldehyde in 0.1 M cacodylate buffer (pH 7.4). After fixation, samples were washed three times for 30 min each in 0.1 M cacodylate buffer, then postfixed with 2% osmium tetroxide in 0.1 M cacodylate buffer at 4°C for 2 hours. The samples were dehydrated in a graded series of ethanol solutions: 50 and 70% ethanol for 30 min each at 4°C, followed by 90% ethanol for 30 min at room temperature, and lastly 100% ethanol three times for 30 min each, then overnight at room temperature. Next, the samples were infiltrated twice with propylene oxide for 30 min each, followed by infiltration in a 70:30 mixture of propylene oxide and resin (Quetol-812, Nisshin EM) for 1 hour. The propylene oxide was allowed to volatilize with the tube lid open overnight. The samples were then transferred to fresh 100% resin and polymerized at 60°C for 48 hours. Semithin sections (1.5 μm thick) were cut using a glass knife on an ultramicrotome (Ultracut UCT, Leica) and stained with 0.5% toluidine blue. Upon reaching the midline of the embryos, ultrathin sections (70 nm thick) were cut using a diamond knife on the ultramicrotome. These sections were mounted on copper grids and stained with 2% uranyl acetate at room temperature for 15 min, washed with distilled water, and subsequently stained with lead stain solution (Sigma-Aldrich) at room temperature for 3 min. The sections were examined using a transmission electron microscope (JEM-1400Plus, JEOL) at an acceleration voltage of 100 kV. Digital images (3296 × 2472 pixels) were captured using a charge-coupled device camera (EM-14830RUBY2, JEOL).

### Explant culture

Dechorionated embryos at approximately 24 ss were placed in L15 medium (11415-064, Thermo Fisher Scientific) and manually excised with 30-gauge needles (Terumo) under a stereomicroscope. For posterior explants, the excision planes along the anterior-posterior axis were around the 21st somite and in the middle of the presomitic mesoderm. For anterior explants, the excisions were made around the 15th and 18th somites. After excision, the fragments were immediately transferred to a 35-mm glass-based or four-chamber glass-based dish (D35C4-20-1.5-N, Cellvis) using a P2 pipette and embedded in 0.5% low–melting point agarose in L15 medium. The explants were then imaged at 28.5°C under the LSM710 confocal microscope.

### Chemical treatment

Dechorionated embryos at 12 ss were soaked in 1/3× Ringer’s solution containing 400 μM NSC23766 (SML0952, Sigma-Aldrich, 50 mM stock in DMSO), 100 μM EHT1864 (S7482, Selleck, 50 mM stock in DMSO), or 500 μM CK666 (ab141231, Abcam, 100 mM stock in DMSO) and incubated at 28°C for 4 hours until fixation with 4% PFA in PBS.

For explant treatment, posterior explants excised from 24-ss *shha:memCherry* embryos were soaked in 400 μM NSC23766 or 20 μM SU5402 (SML0443, Sigma-Aldrich, 50 mM stock in DMSO) diluted in L15 medium and incubated at 28°C for 1 hour. The explants were then embedded in 0.5% low–melting point agarose in L15 medium containing either 400 μM NSC23766 or 20 μM SU5402 in a four-chamber glass-based dish and subjected to time-lapse imaging under the LSM710 confocal microscope.

For blebbistatin treatment, *actb2:memCherry* embryos at 18 ss were soaked in 100 or 200 μM (−)-blebbistatin (021-17041, Wako, 10 mM stock in DMSO) and incubated at 26°C for 4 hours. The embryos were then subjected to EdU incorporation experiments, with EdU solution containing the corresponding concentration of blebbistatin.

### Quantification

All graphs with statistics (Student’s *t* test) were generated using Prism (GraphPad).

#### Distance from the tailbud after photoconversion

The distance between photoconverted cells in embryos labeled with *nuc-KikGR* mRNA and the anterior edge of the CNH was manually measured using the Line and Measurement tools in Fiji.

#### Cell velocity measurement from kymographs

After loading time-lapse images of explants in Fiji, segmented lines were manually drawn along FP or HC using the Line tool to identify their lateral cell edges and defined as regions of interest (ROIs). These ROIs were used to generate kymographs using the KymographBuilder plugin. The angles of the lines shown in the kymographs were manually measured using the Line and Measurement tools, which were then used to calculate cell velocity.

#### Angles of cell lateral edges

Angles between the lateral and basal edges of FP and HC cells were manually measured using the Angle tool in Fiji. The anterior, middle, and posterior regions were defined as the field of view under the LSM710 confocal microscope with 40× objective (corresponding to approximately 106 μm width along the anterior-posterior axis), located 500, 250, and 0 μm away from the CNH, respectively.

#### Actin distribution along the apicobasal axis of the posterior lateral cell membrane

For this analysis, *actb2:memCherry* embryos mosaically injected with *Actin-Chromobody-GFP* mRNA at the eight-cell stage were used. Fluorescence intensity of *Actin-Chromobody-GFP* signals along the posterior lateral cell membrane of FP and HC cells was measured using the Segmented Line tool in Fiji. If *Actin-Chromobody-GFP*–positive cells were arranged consecutively, the posterior lateral cell membrane of the posterior-most cell was analyzed. Signal intensities from the apical and basal 30% of the membrane in the obtained plot profiles were summed separately, and the apical/basal signal ratio was calculated.

#### Relative position of the Golgi apparatus along the anterior-posterior axis within a cell

The length *L* of the apical surface of an FP or HC cell was manually measured using the Line tool in Fiji. The distance from the anterior end of the cell to the anterior edge of the Golgi apparatus highlighted by mNeonGreen-Giantin was measured (denoted as *a*), and the distance from the anterior end of the cell to the posterior edge of the Golgi apparatus was measured as *b*. The relative Golgi position along the anterior-posterior axis was defined as (*a* + *b*)/2*L*. Because the values *a* and *b* fall between 0 and *L*, this index takes a value between 0 and 1. A larger index value indicates that the Golgi apparatus is located more posteriorly in the cell. The anterior, middle, and posterior regions were defined as the field of view under the LSM710 confocal microscope with 40× objective (corresponding to approximately 106 μm width along the anterior-posterior axis), located 500, 250, and 0 μm away from the CNH, respectively.

#### EdU-positive rate

The numbers of nuclei (highlighted by anti-Sox2 staining) and EdU-positive cells within a field of view were manually counted in Fiji and used to calculate the EdU-positive rate. The anterior, middle, and posterior regions were defined as the field of view under the LSM710 confocal microscope with 40× objective (corresponding to approximately 106 μm width along the anterior-posterior axis), located 500, 250, and 0 μm away from the CNH, respectively.

#### Cell length

For Fig. 3C, cell length was defined as the length of the cell edge opposite to the side in contact with the ECM, measured along the anterior-posterior axis. For Fig. 3F, we used the distance between the nuclei of two adjacent cells as a proxy for cell length. Specifically, we measured the distance between nuclei that were positive for anti-Sox2 staining along the anterior-posterior axis within the FP or HC. These measurements were manually performed using the Line tool in Fiji.

#### Yap nuclear/cytoplasmic ratio

The nuclear/cytoplasmic ratio of Yap localization was calculated based on manual measurement of the fluorescence intensity within each cell of the confocal images by drawing ROIs within either the nucleus or the cytoplasm of the cell using the Oval tool in Fiji.

#### Fluorescence intensity of HCR images along the anterior-posterior axis

For fig. S4, segmented lines were manually drawn along the FP or HC using the Line tool in Fiji, set to a width of 10. The signal intensity along the lines was calculated using the Plot Profile tool and exported to Prism for statistics.

For fig. S9, because the imaged region spanned a much longer anterior-posterior distance, the midline tissues extended across multiple *z* planes in the confocal stack. To enable quantitative analysis, all ROIs containing midline tissue from different *z* planes were merged into a single projection image using a custom ImageJ JavaScript script. To correct for potential depth-dependent variations in fluorescence intensity over the long imaging range, the HCR signal was normalized to the mCherry signal derived from the *shha:memCherry* transgene, which is ubiquitously expressed along the anterior-posterior axis. Before normalization, membrane-localized mCherry peaks were excluded using the minimum_filter1d function (Python SciPy library) to extract the baseline signal. The normalized signal was then divided into 20 equal bins along the anterior-posterior axis. For each bin, the average fluorescence intensity was calculated and subsequently rescaled so that the maximum value across bins was set to 1.

### Segmentation and 3D rendering

A confocal z-stack image of a *shha:memCherry* embryo at 22 ss showing cell membrane signals of the midline tissues was processed using a 3D median filter (radius = 2 pixels) in Fiji. This was followed by cell segmentation with Cellpose (*66*) using the following parameters: flow threshold = 0.8, cell probability = −0.6 to 0, and diameter = 45.6. Corresponding mask images were generated as TIFF files for each cell. Cells that were not adequately detected by Cellpose were manually masked using the Segmentation Filter plugin in Fiji. The mask images were then converted to STL files using the ImageMesh function based on the dual marching cubes algorithm in Mathematica 14.0 (Wolfram Research). Rendering was performed with the Cycles engine in Blender 3.0.0 (Blender Foundation).

### Vertex model for tissue elongation

We modeled each cell as a quadrilateral within a vertex framework to investigate the elongation of aligned midline tissues, which is driven by cell division and migration. Let $V=\{r_{j}=\left(x_{j},y_{j}\right)\;|\;j=1,2,\cdots ,n\}$ be the set of vertices, and let $C=\{C_{i}|i=1,2,\cdots ,m\}$ be the set of cells, where each cell is defined by four vertices from V . Here, n denotes the number of vertices and m indicates the number of cells. In the vertex model, adjacent cells $C_{i}$ and $C_{j}$ share vertices along their mutual boundary (see fig. S5 for a schematic diagram of the model). In our numerical simulations, the x coordinates of the two anterior-most vertices are fixed.

The motion of each vertex is governed by a gradient descent on an energy function U , where each vertex moves in the direction that minimizes U

$$\begin{array}{c} \begin{array}{c} U=\frac{a}{2}\sum_{i=1}^{m}{\left[V_{i}\left(t\right)-V_{0}\right]}^{2}+\frac{b}{2}\sum_{i=1}^{m}L_{i}^{2}+c\sum_{i=1}^{m}\sum_{r_{j}\in B_{i}}f\left(|r_{j}-p_{j}|\right)\\ \;+d\sum_{i=1}^{m}\sum_{r_{j}\in A_{i}}g\left(|r_{j}-p_{j}|\right) \end{array} \end{array}$$

where a , b , c , and d are positive constants. The first term represents area conservation, with $V_{i}\left(t\right)$ as the area of cell $C_{i}$ and $V_{0}$ as the target cell area. The second term represents cell perimeter contraction, where $L_{i}$ is the perimeter of $C_{i}$ . The third term represents adhesion between the basal surface and the ECM, where $B_{i}$ contains the two basal vertices of $C_{i}$ , and vertex $p_{j}$ is defined by $p_{j}=\left(x_{j},0\right)$ . The function f(x) is defined as follows: $f\left(x\right)={\left(x-h\right)}^{2}\;/2-{\left(h_{\text{th}}-h\right)}^{2}\;/4$ for $0\leq x<\left(h_{\text{th}}+h\right)\;/\;2$ ; $f\left(x\right)=-{\left(x-h_{\text{th}}\right)}^{2}\;/2$ for $\left(h_{\text{th}}+h\right)\;/\;2\leq x<h_{\text{th}}$ ; and f(x)=0 for $x\geq h_{\text{th}}$ , where h is the ECM position and $h_{\text{th}}$ is the adhesion threshold for basal vertices relative to the ECM. The fourth term represents apical surface repulsion from the external structures, with $A_{i}$ containing the two apical vertices of $C_{i}$ . The function g(x) is defined as g(x)=0 for x<ℓ and $g\left(x\right)={\left(x-\ell \right)}^{2}\;/2$ for x≥ℓ , where ℓ denotes the position of the external structure.

The force on vertex $r_{j}$ is given by $F_{j}=-\delta U\;/\delta r_{j}$ , and vertex motion follows

$$\tau \frac{dr_{j}}{\mathrm{dt}}=F_{j}+\sigma \xi \left(t\right)$$

where τ>0 is a viscosity coefficient, ξ(t) is white noise, and σ>0 is the noise amplitude.

Cell division is regulated by elastic energy in the apical and basal edges. Let $U_{i}=b\left(L_{i,\text{apical}}^{2}+L_{i,\text{basal}}^{2}\right)/2$ , where $L_{i,\text{apical}}$ and $L_{i,\text{basal}}$ are the lengths of apical and basal edges of $C_{i}$ , respectively. If $U_{i}$ exceeds a threshold $U_{\text{th}}>0$ at time $T_{i}$ , and a specified time $S_{i}$ elapsed (i.e., $t>T_{i}+S_{i}$ ), then $C_{i}$ divides to two daughter cells $C_{i^{\prime }}$ and $C_{i^{\prime \prime }}$ , using midpoints of the apical and basal edges (see fig. S7A).

To model migration, we introduced an external migration force. Let $v_{i}=v_{i}\left(t\right)$ be the migration force of $C_{i}$ at time t , governed by

$$\eta \frac{dv_{i}}{\mathrm{dt}}=-v_{i}+w_{i}\left(t\right)$$

where η>0 is a time constant and the initial value of $v_{i}$ is 0 . After cell division, both daughter cells $C_{i^{\prime }}$ and $C_{i^{\prime \prime }}$ start with $v_{i^{\prime }}=0$ and $v_{i^{\prime \prime }}=0$ , respectively. The migration activity $w_{i}$ is defined by

$$w_{i}\left(t\right)=v_{\text{max}}{\left[\frac{x_{i}\left(t\right)-x_{\text{min}}}{x_{\text{max}}-x_{\text{min}}}\right]}^{\alpha }$$

where $x_{i}\left(t\right)$ is the x coordinate of the centroid of $C_{i}$ , and $x_{\text{max}}=\text{max}$$\{x_{i}\left(t\right)|i=1,2,\;\cdots \;,m\}$ and $x_{\text{min}}=\text{min}\{x_{i}\left(t\right)\;|i=1,2,\;\cdots \;,m\}$ are the maximum and minimum centroids along the x axis, respectively. $v_{\text{max}}$ denotes the maximum of the external migration force. The exponent α>0 controls the convexity of the gradient shape: α = 0.01 limits migration to the most posterior cell (no collective migration), α = 100 induces uniform migration across cells, and α = 1 results in graded collective migration. The force $v_{k}$ is applied to the posterior basal vertex of $C_{k}$ . The governing equations were solved using the Euler-Maruyama method for vertex dynamics and the Runge-Kutta method for migration forces, with a time step of dt=0.001 . The initial number of cells was set to m=32 . While the linear gradient ( α = 1) was used for graded migration in our simulations in the Results section, unbiased cell proliferation was also observed for a range of α values (0.85 to 1.5; see Supplementary Text), indicating that distributed cell proliferation is robust to gradient shape in our model and can accommodate a variety of gradient shapes beyond a strictly linear distribution. The values of other parameters used in the vertex model are listed in table S1.

In simulations of complex midline tissues consisting of FP, HC, and notochord cells, FP and HC cells follow the same vertex model described above. Notochord cells are modeled similarly, incorporating area conservation, perimeter contraction, and ECM adhesion. In the initial state, the number of notochord cells is set to twice that of FP or HC cells (see Fig. 5, B and C). The notochord elongates via cell division, with the division time $D_{i}$ specified for the i th notochord cell. In Fig. 5 (B and C), $D_{i}$ was set to 0.8 and 1.2 , respectively. In cases of modeling adhesion between the posterior-most FP cell and the notochord, the basal posterior vertex of the posterior-most FP cell and the upper posterior vertex of the posterior-most notochord cell are treated as a shared vertex. Adhesion between HC and notochord cells is modeled similarly.

## References

[1] J. R. Vieira, B. Shah, S. Dupraz, I. Paredes, P. Himmels, G. Schermann, H. Adler, A. Motta, L. Gärtner, A. Navarro-Aragall, E. Ioannou, E. Dyukova, R. Bonnavion, A. Fischer, D. Bonanomi, F. Bradke, C. Ruhrberg, C. Ruiz de Almodóvar, Endothelial PlexinD1 signaling instructs spinal cord vascularization and motor neuron development. Neuron 110, 4074–4089.e6 (2022). 36549270 10.1016/j.neuron.2022.12.005 PMC9796814

[2] B. Bénazéraf, M. Beaupeux, M. Tchernookov, A. Wallingford, T. Salisbury, A. Shirtz, A. Shirtz, D. Huss, O. Pourquié, P. François, R. Lansford, Multi-scale quantification of tissue behavior during amniote embryo axis elongation. Development 144, 4462–4472 (2017). 28835474 10.1242/dev.150557 PMC13471720

[3] F. Xiong, W. Ma, B. Bénazéraf, L. Mahadevan, O. Pourquié, Mechanical coupling coordinates the co-elongation of axial and paraxial tissues in avian embryos. Dev. Cell 55, 354–366.e5 (2020). 32918876 10.1016/j.devcel.2020.08.007 PMC7685225

[4] D. Bornhorst, P. Xia, H. Nakajima, C. Dingare, W. Herzog, V. Lecaudey, N. Mochizuki, C.-P. Heisenberg, D. Yelon, S. Abdelilah-Seyfried, Biomechanical signaling within the developing zebrafish heart attunes endocardial growth to myocardial chamber dimensions. Nat. Commun. 10, 4113 (2019). 31511517 10.1038/s41467-019-12068-x PMC6739419

[5] Y. Takahashi, R. Kudo, R. Tadokoro, Y. Atsuta, Coordination between body growth and tissue growth: Wolffian duct elongation and somitogenesis proceed in harmony with axial growth. Int. J. Dev. Biol. 62, 79–84 (2018). 29616742 10.1387/ijdb.170290yt

[6] R. Keller, Mechanisms of elongation in embryogenesis. Development 133, 2291–2302 (2006). 16720874 10.1242/dev.02406

[7] A. Boutillon, S. P. Banavar, O. Campàs, Conserved physical mechanisms of cell and tissue elongation. Development 151, dev202687 (2024). 38767601 10.1242/dev.202687 PMC11190436

[8] M. Freeman, Feedback control of intercellular signalling in development. Nature 408, 313–319 (2000). 11099031 10.1038/35042500

[9] A. D. Lander, Pattern, growth and control. Cell 144, 955–969 (2011). 21414486 10.1016/j.cell.2011.03.009 PMC3128888

[10] Y. E. Antebi, N. Nandagopal, M. B. Elowitz, An operational view of intercellular signaling pathways. Curr. Opin. Syst. Biol. 1, 16–24 (2017). 29104946 10.1016/j.coisb.2016.12.003 PMC5665397

[11] Y. Tan, *Handbook of Research on Design, Control, and Modeling of Swarm Robotics* (IGI Global, 2016).

[12] M. Rubenstein, A. Cornejo, R. Nagpal, Programmable self-assembly in a thousand-robot swarm. Science 345, 795–799 (2014). 25124435 10.1126/science.1254295

[13] M. Senanayake, I. Senthooran, J. C. Barca, H. Chung, J. Kamruzzaman, M. Murshed, Search and tracking algorithms for swarms of robots: A survey. Rob. Auton. Syst. 75, 422–434 (2016).

[14] S. B. P. McLaren, B. J. Steventon, Anterior expansion and posterior addition to the notochord mechanically coordinate zebrafish embryo axis elongation. Development 148, dev199459 (2021). 34086031 10.1242/dev.199459 PMC8327291

[15] W. S. Talbot, B. Trevarrow, M. E. Halpern, A. E. Melby, G. Farr, J. H. Postlethwait, T. Jowett, C. B. Kimmel, D. Kimelman, A homeobox gene essential for zebrafish notochord development. Nature 378, 150–157 (1995). 7477317 10.1038/378150a0

[16] K. Ellis, J. Bagwell, M. Bagnat, Notochord vacuoles are lysosome-related organelles that function in axis and spine morphogenesis. J. Cell Biol. 200, 667–679 (2013). 23460678 10.1083/jcb.201212095 PMC3587825

[17] J. Bagwell, J. Norman, K. Ellis, B. Peskin, J. Hwang, X. Ge, S. V. Nguyen, S. K. McMenamin, D. Y. Stainier, M. Bagnat, Notochord vacuoles absorb compressive bone growth during zebrafish spine formation. eLife 9, e51221 (2020). 31995030 10.7554/eLife.51221 PMC7012607

[18] D. Kimelman, N. L. Smith, J. K. H. Lai, D. Y. Stainier, Regulation of posterior body and epidermal morphogenesis in zebrafish by localized Yap1 and Wwtr1. eLife 6, e31065 (2017). 29283341 10.7554/eLife.31065 PMC5773182

[19] R. H. Row, S. R. Tsotras, H. Goto, B. L. Martin, The zebrafish tailbud contains two independent populations of midline progenitor cells that maintain long-term germ layer plasticity and differentiate based on local signaling cues. Development 143, 244–254 (2015). 26674311 10.1242/dev.129015 PMC4725346

[20] T. Dheen, I. Sleptsova-Friedrich, Y. Xu, M. Clark, H. Lehrach, Z. Gong, V. Korzh, Zebrafish tbx-c functions during formation of midline structures. Development 126, 2703–2713 (1999). 10331981 10.1242/dev.126.12.2703

[21] A.-G. Borycki, L. Mendham, C. P. Emerson, Control of somite patterning by Sonic hedgehog and its downstream signal response genes. Development 125, 777–790 (1998). 9435297 10.1242/dev.125.4.777

[22] J. P. Amorim, A. Gali-Macedo, H. Marcelino, R. Bordeira-Carriço, S. Naranjo, S. Rivero-Gil, J. Teixeira, M. Galhardo, J. Marques, J. Bessa, A conserved notochord enhancer controls pancreas development in vertebrates. Cell Rep. 32, 107862 (2020). 32640228 10.1016/j.celrep.2020.107862 PMC7355232

[23] L. Lleras Forero, R. Narayanan, L. F. Huitema, M. VanBergen, A. Apschner, J. Peterson-Maduro, I. Logister, G. Valentin, L. G. Morelli, A. C. Oates, S. Schulte-Merker, Segmentation of the zebrafish axial skeleton relies on notochord sheath cells and not on the segmentation clock. eLife 7, e33843 (2018). 29624170 10.7554/eLife.33843 PMC5962341

[24] J. Briscoe, J. Ericson, The specification of neuronal identity by graded sonic hedgehog signalling. Semin. Cell Dev. Biol. 10, 353–362 (1999). 10441550 10.1006/scdb.1999.0295

[25] O. Cleaver, P. A. Krieg, VEGF mediates angioblast migration during development of the dorsal aorta in *Xenopus*. Development 125, 3905–3914 (1998). 9729498 10.1242/dev.125.19.3905

[26] R. Esterberg, J.-M. Delalande, A. Fritz, Tailbud-derived Bmp4 drives proliferation and inhibits maturation of zebrafish chordamesoderm. Development 135, 3891–3901 (2008). 18948415 10.1242/dev.029264 PMC2765817

[27] J. P. Kanki, R. K. Ho, The development of the posterior body in zebrafish. Development 124, 881–893 (1997). 9043069 10.1242/dev.124.4.881

[28] M. J. Harrington, K. Chalasani, R. Brewster, Cellular mechanisms of posterior neural tube morphogenesis in the zebrafish. Dev. Dyn. 239, 747–762 (2010). 20077475 10.1002/dvdy.22184

[29] R. L. Davis, M. W. Kirschner, The fate of cells in the tailbud of *Xenopus laevis*. Development 127, 255–267 (2000). 10603344 10.1242/dev.127.2.255

[30] B. L. Martin, D. Kimelman, Canonical Wnt signaling dynamically controls multiple stem cell fate decisions during vertebrate body formation. Dev. Cell 22, 223–232 (2012). 22264734 10.1016/j.devcel.2011.11.001 PMC3465166

[31] A. Steffen, M. Ladwein, G. A. Dimchev, A. Hein, L. Schwenkmezger, S. Arens, K. I. Ladwein, J. M. Holleboom, F. Schur, J. V. Small, J. Schwarz, R. Gerhard, J. Faix, T. E. B. Stradal, C. Brakebusch, K. Rottner, Rac function is critical for cell migration but not required for spreading and focal adhesion formation. J. Cell Sci. 126, 4572–4588 (2013). 23902686 10.1242/jcs.118232 PMC3817791

[32] P. R. Carney, E. Couve, Cell polarity changes and migration during early development of the avian peripheral auditory system. Anat. Rec. 225, 156–164 (1989). 2817430 10.1002/ar.1092250211

[33] J. Magdalena, T. H. Millard, L. M. Machesky, Microtubule involvement in NIH 3T3 Golgi and MTOC polarity establishment. J. Cell Sci. 116, 743–756 (2003). 12538774 10.1242/jcs.00288

[34] B. T. Schaar, S. K. McConnell, Cytoskeletal coordination during neuronal migration. Proc. Natl. Acad. Sci. 102, 13652–13657 (2005). 16174753 10.1073/pnas.0506008102 PMC1199551

[35] N. Hino, L. Rossetti, A. Marín-Llauradó, K. Aoki, X. Trepat, M. Matsuda, T. Hirashima, ERK-mediated mechanochemical waves direct collective cell polarization. Dev. Cell 53, 646–660.e8 (2020). 32497487 10.1016/j.devcel.2020.05.011

[36] N. L. Nerurkar, C. Lee, L. Mahadevan, C. J. Tabin, Molecular control of macroscopic forces drives formation of the vertebrate hindgut. Nature 565, 480–484 (2019). 30651642 10.1038/s41586-018-0865-9 PMC6397660

[37] B. Bénazéraf, P. Francois, R. E. Baker, N. Denans, C. D. Little, O. Pourquié, A random cell motility gradient downstream of FGF controls elongation of an amniote embryo. Nature 466, 248–252 (2010). 20613841 10.1038/nature09151 PMC3118990

[38] K. Tsujita, T. Itoh, “Membrane tension and mechanobiology of cell migration,” in *Plasma Membrane Shaping* (Elsevier, 2023), pp. 281–293.

[39] M. Aragona, A. Sifrim, M. Malfait, Y. Song, J. Van Herck, S. Dekoninck, S. Gargouri, G. Lapouge, B. Swedlund, C. Dubois, P. Baatsen, K. Vints, S. Han, F. Tissir, T. Voet, B. D. Simons, C. Blanpain, Mechanisms of stretch-mediated skin expansion at single-cell resolution. Nature 584, 268–273 (2020). 32728211 10.1038/s41586-020-2555-7 PMC7116042

[40] J. McGinn, A. Hallou, S. Han, K. Krizic, S. Ulyanchenko, R. Iglesias-Bartolome, F. J. England, C. Verstreken, K. J. Chalut, K. B. Jensen, B. D. Simons, M. P. Alcolea, A biomechanical switch regulates the transition towards homeostasis in oesophageal epithelium. Nat. Cell Biol. 23, 511–525 (2021). 33972733 10.1038/s41556-021-00679-w PMC7611004

[41] W. Kowalczyk, L. Romanelli, M. Atkins, H. Hillen, C. Bravo González-Blas, J. Jacobs, J. Xie, S. Soheily, E. Verboven, I. M. Moya, S. Verhulst, M. de Waegeneer, L. Sansores-Garcia, L. van Huffel, R. L. Johnson, L. A. van Grunsven, S. Aerts, G. Halder, Hippo signaling instructs ectopic but not normal organ growth. Science 378, eabg3679 (2022). 36395225 10.1126/science.abg3679

[42] K. Gnedeva, A. Jacobo, J. D. Salvi, A. A. Petelski, A. J. Hudspeth, Elastic force restricts growth of the murine utricle. eLife 6, e25681 (2017). 28742024 10.7554/eLife.25681 PMC5550282

[43] J. B. Miesfeld, B. A. Link, Establishment of transgenic lines to monitor and manipulate Yap/Taz-Tead activity in zebrafish reveals both evolutionarily conserved and divergent functions of the Hippo pathway. Mech. Dev. 133, 177–188 (2014). 24560909 10.1016/j.mod.2014.02.003 PMC4138299

[44] X. Serra-Picamal, V. Conte, R. Vincent, E. Anon, D. T. Tambe, E. Bazellieres, J. P. Butler, J. J. Fredberg, X. Trepat, Mechanical waves during tissue expansion. Nat. Phys. 8, 628–634 (2012).

[45] R. W. Beard, J. Lawton, F. Y. Hadaegh, A coordination architecture for spacecraft formation control. IEEE Trans Control Syst Technol 9, 777–790 (2001).

[46] D. P. Scharf, F. Y. Hadaegh, S. R. Ploen, “A survey of spacecraft formation flying guidance and control. Part II: Control,” paper presented at Proceedings of the American Control Conference, Boston, MA, USA, 30 June to 2 July 2004.

[47] K. Ishimatsu, T. W. Hiscock, Z. M. Collins, D. W. K. Sari, K. Lischer, D. L. Richmond, Y. Bessho, T. Matsui, S. G. Megason, Size-reduced embryos reveal a gradient scaling based mechanism for zebrafish somite formation. Development 145, dev161257 (2018). 29769221 10.1242/dev.161257 PMC6031319

[48] M. F. Simsek, E. M. Özbudak, Spatial fold change of FGF signaling encodes positional information for segmental determination in zebrafish. Cell Rep. 24, 66–78.e8 (2018). 29972792 10.1016/j.celrep.2018.06.023 PMC6063364

[49] F. Xiong, W. Ma, T. W. Hiscock, K. R. Mosaliganti, A. R. Tentner, K. A. Brakke, N. Rannou, A. Gelas, L. Souhait, I. A. Swinburne, N. D. Obholzer, S. G. Megason, Interplay of cell shape and division orientation promotes robust morphogenesis of developing epithelia. Cell 159, 415–427 (2014). 25303534 10.1016/j.cell.2014.09.007 PMC4273647

[50] J. Shin, J. Chen, L. Solnica-Krezel, Efficient homologous recombination-mediated genome engineering in zebrafish using TALE nucleases. Development 141, 3807–3818 (2014). 25249466 10.1242/dev.108019 PMC4197590

[51] C. B. Kimmel, W. W. Ballard, S. R. Kimmel, B. Ullmann, T. F. Schilling, Stages of embryonic development of the zebrafish. Dev. Dyn. 203, 253–310 (1995). 8589427 10.1002/aja.1002030302

[52] D. G. Gibson, L. Young, R.-Y. Chuang, J. C. Venter, C. A. Hutchison III, H. O. Smith, Enzymatic assembly of DNA molecules up to several hundred kilobases. Nat. Methods 6, 343–345 (2009). 19363495 10.1038/nmeth.1318

[53] A. Shkumatava, S. Fischer, F. Müller, U. Strahle, C. J. Neumann, Sonic hedgehog, secreted by amacrine cells, acts as a short-range signal to direct differentiation and lamination in the zebrafish retina. Development 131, 3849–3858 (2004). 15253932 10.1242/dev.01247

[54] A. Munjal, E. Hannezo, T. Y.-C. Tsai, T. J. Mitchison, S. G. Megason, Extracellular hyaluronate pressure shaped by cellular tethers drives tissue morphogenesis. Cell 184, 6313–6325.e18 (2021). 34942099 10.1016/j.cell.2021.11.025 PMC8722442

[55] S. A. Harvey, S. Tümpel, J. Dubrulle, A. F. Schier, J. C. Smith, No tail integrates two modes of mesoderm induction. Development 137, 1127–1135 (2010). 20215349 10.1242/dev.046318 PMC2835328

[56] K. Kawakami, H. Takeda, N. Kawakami, M. Kobayashi, N. Matsuda, M. Mishina, A transposon-mediated gene trap approach identifies developmentally regulated genes in zebrafish. Dev. Cell 7, 133–144 (2004). 15239961 10.1016/j.devcel.2004.06.005

[57] A. O. Chertkova, M. Mastop, M. Postma, N. van Bommel, S. van der Niet, K. L. Batenburg, L. Joosen, T. W. J. Gadella Jr., Y. Okada, J. Goedhart, Robust and bright genetically encoded fluorescent markers for highlighting structures and compartments in mammalian cells. bioRxiv 160374 [Preprint] (2020). 10.1101/160374.

[58] I. A. Swinburne, K. R. Mosaliganti, A. A. Green, S. G. Megason, Improved long-term imaging of embryos with genetically encoded α-bungarotoxin. PLOS ONE 10, e0134005 (2015). 26244658 10.1371/journal.pone.0134005 PMC4526548

[59] K. Skouloudaki, M. Puetz, M. Simons, J.-R. Courbard, C. Boehlke, B. Hartleben, C. Engel, M. J. Moeller, C. Englert, F. Bollig, T. Schäfer, H. Ramachandran, M. Mlodzik, T. B. Huber, E. W. Kuehn, E. Kim, A. Kramer-Zucker, G. Walz, Scribble participates in Hippo signaling and is required for normal zebrafish pronephros development. Proc. Natl. Acad. Sci. U.S.A. 106, 8579–8584 (2009). 19439659 10.1073/pnas.0811691106 PMC2688978

[60] Z. Lele, A. Folchert, M. Concha, G.-J. Rauch, R. Geisler, F. Rosa, S. W. Wilson, M. Hammerschmidt, L. Bally-Cuif, Parachute/n-cadherin is required for morphogenesis and maintained integrity of the zebrafish neural tube. Development 129, 3281–3294 (2002). 12091300 10.1242/dev.129.14.3281

[61] S. G. Megason, In toto imaging of embryogenesis with confocal time-lapse microscopy. Methods Mol. Biol. 546, 317–332 (2009). 19378112 10.1007/978-1-60327-977-2_19 PMC2826616

[62] S. Daetwyler, C. D. Modes, R. Fiolka, Fiji plugin for annotating movies with custom arrows. Biol. Open 9, bio056200 (2020). 33168591 10.1242/bio.056200 PMC7725597

[63] J. Schindelin, I. Arganda-Carreras, E. Frise, V. Kaynig, M. Longair, T. Pietzsch, S. Preibisch, C. Rueden, S. Saalfeld, B. Schmid, J.-Y. Tinevez, D. J. White, V. Hartenstein, K. Eliceiri, P. Tomancak, A. Cardona, Fiji: An open-source platform for biological-image analysis. Nat. Methods 9, 676–682 (2012). 22743772 10.1038/nmeth.2019 PMC3855844

[64] T. Kawanishi, T. Kaneko, Y. Moriyama, M. Kinoshita, H. Yokoi, T. Suzuki, A. Shimada, H. Takeda, Modular development of the teleost trunk along the dorsoventral axis and zic1/zic4 as selector genes in the dorsal module. Development 140, 1486–1496 (2013). 23462471 10.1242/dev.088567

[65] H. M. T. Choi, M. Schwarzkopf, M. E. Fornace, A. Acharya, G. Artavanis, J. Stegmaier, A. Cunha, N. A. Pierce, Third-generation in situ hybridization chain reaction: Multiplexed, quantitative, sensitive, versatile, robust. Development 145, dev165753 (2018). 29945988 10.1242/dev.165753 PMC6031405

[66] C. Stringer, T. Wang, M. Michaelos, M. Pachitariu, Cellpose: A generalist algorithm for cellular segmentation. Nat. Methods 18, 100–106 (2021). 33318659 10.1038/s41592-020-01018-x

