# Supplementary material for: Formation control between leader and migratory follower tissues allows coordinated growth
Source: Sci Adv. 2025 Jul 30;11(31):eads2310. doi: 10.1126/sciadv.ads2310 (PMC12309693; doi:10.1126/sciadv.ads2310)
Supplement: Supplementary file 1 — Figs. S1 to S9 Supplementary Text Table S1 Legends for movies S1 to S12 [file sciadv.ads2310_sm.pdf]

Science Advances
AAAS

Supplementary Materials for

Formation control between leader and migratory follower tissues allows coordinated growth

Toru Kawanishi *et al.*

Corresponding author: Toru Kawanishi, toru.kawanishi@life.isct.ac.jp;
Sean G. Megason, megason@hms.harvard.edu

Sci. Adv. **11**, eads2310 (2025)
DOI: 10.1126/sciadv.ads2310

The PDF file includes:

Figs. S1 to S9
Supplementary Text
Table S1
Legends for movies S1 to S12

Other Supplementary Material for this manuscript includes the following:

Movies S1 to S12

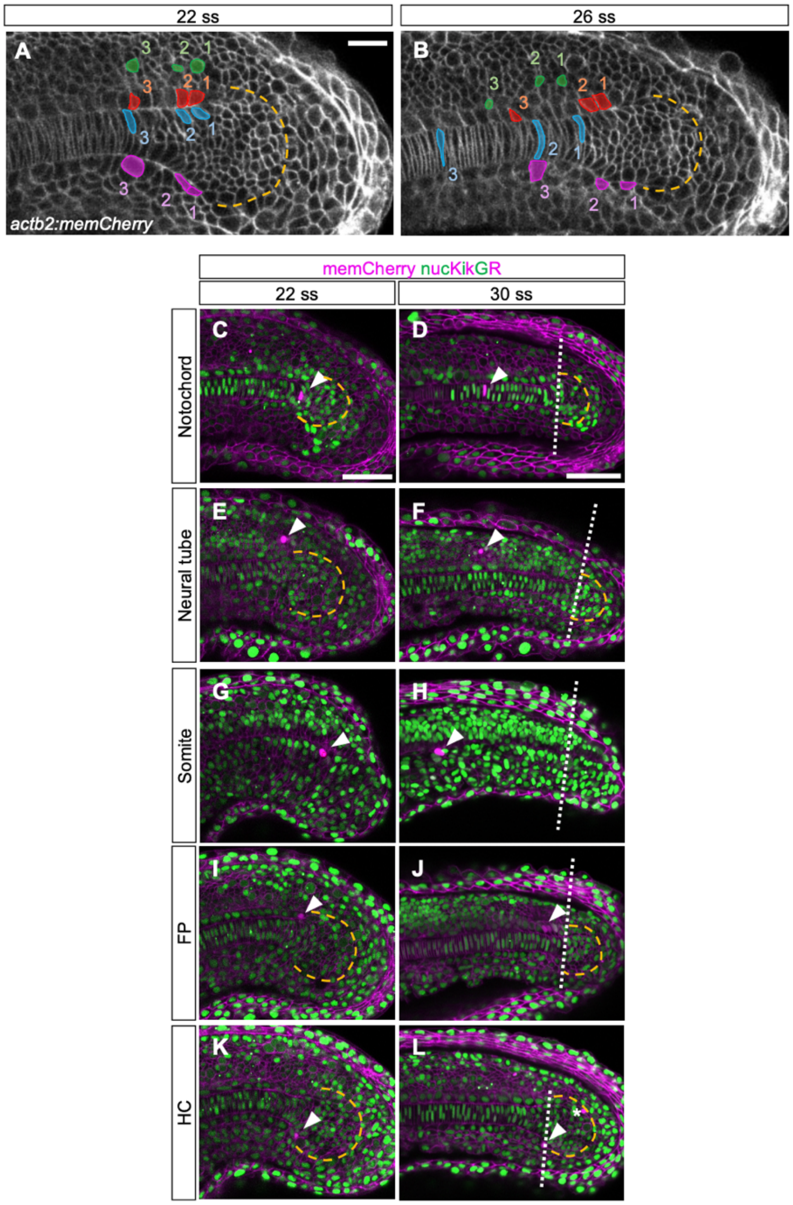

Fig. S1. Displacement of cells in the tailbud during body axis elongation.

(**A, B**) Confocal time-lapse images of a zebrafish tailbud expressing memCherry. Individual cells of the neural tube, FP, notochord and HC are numbered and delineated in green, red, blue and magenta, respectively. Dashed lines delineate the CNH.

(**C-L**) Photoconverted cells of the notochord (C, D), neural tube (E, F), somite (G, H), FP (I, J) and HC (K, L) in the tailbud expressing nuclear KikGR and memCherry at 22 ss (just after photoconversion) and 30 ss (4 hours later). Orange dashed lines delineate the CNH. White dotted lines indicate the positions of the cells along the anterior-posterior axis when they are photoconverted.

Scale bars, 20 µm (A), 50 µm (C, D).

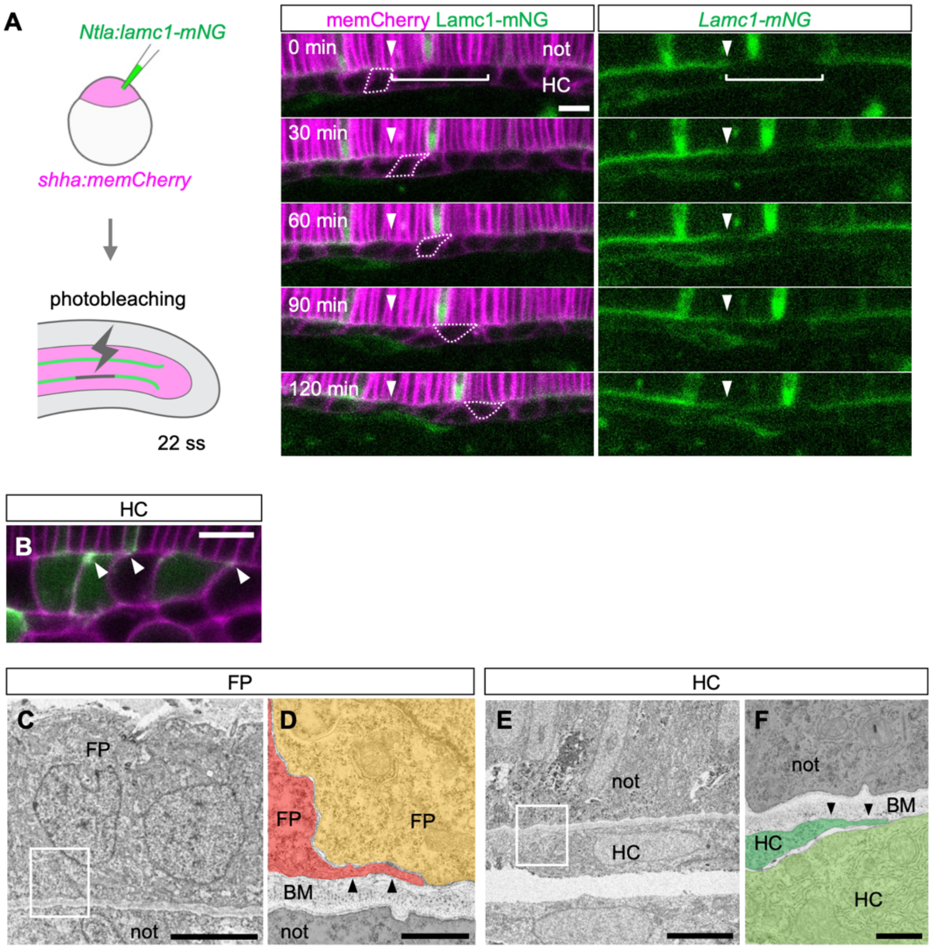

Fig. S2. Posterior migration of the FP and HC cells.

(**A**) Time-lapse images of HC cells after photobleaching of laminin c1-mNeonGreen (brackets). A single HC representative cell is delineated by dashed lines. The anterior boundary of the photobleached region is indicated by arrowheads. Anterior to the left.
(**B**) Posterior HC cells expressing Actin-Chromobody-GFP mosaically and memCherry. Arrowheads indicate GFP accumulation at the posterior side of the cells. Anterior to the left.
(**C-F**) Electron micrographs of FP (C, D) and HC (E, F) in the tail region. White boxes in (C) and (E) indicate the regions corresponding to the magnified views in (D) and (F), respectively, where individual cells are pseudocolored. Arrowheads indicate posteriorly directed protrusions of FP and HC cells on the basement membrane (BM).
Scale bars, 10 µm (A, B), 5 µm (C, E), 1 µm (D, F).

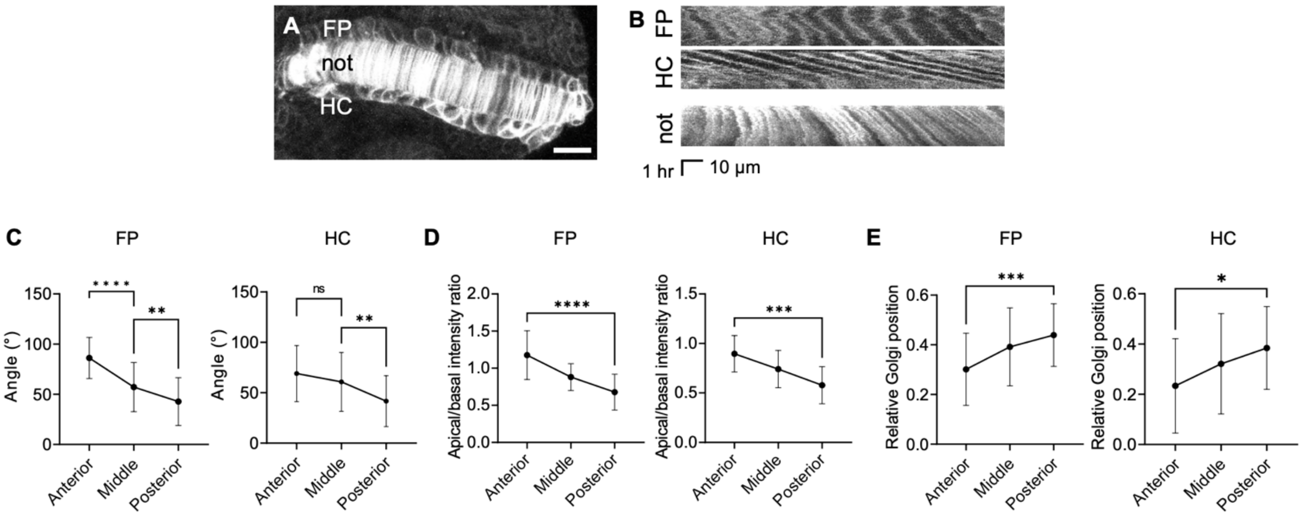

Fig. S3. Collective migration of FP and HC cells ex vivo.

(**A**) Lateral view of a cultured posterior explant from a *shha:memCherry* embryo.
(**B**) Kymographs of FP, HC and notochord cells in a posterior explant.
(**C**) Cell obliqueness measured by the posterior basal angle within FP and HC cells along the anterior-posterior axis (FP: $N = 3$ embryos, $n = 46$ cells for anterior; $N = 3$ embryos, $n = 80$ cells for middle; $N = 3$ embryos, $n = 66$ cells for posterior. HC: $N = 3$ embryos, $n = 23$ cells for anterior; $N = 3$ embryos, $n = 46$ cells for middle; $N = 3$ embryos, $n = 29$ cells for posterior).
(**D**) Actin distribution quantified as the apical/basal actin accumulation ratio within FP and HC cells along the anterior-posterior axis (FP: $N = 8$ embryos, $n = 25$ cells for anterior; $N = 5$ embryos, $n = 11$ cells for middle; $N = 5$ embryos, $n = 66$ cells for posterior. HC: $N = 6$ embryos, $n = 9$ cells for anterior; $N = 7$ embryos, $n = 13$ cells for middle; $N = 9$ embryos, $n = 18$ cells for posterior).
(**E**) Relative anterior-posterior position of the Golgi apparatus within FP and HC cells along the anterior-posterior axis (FP: $N = 3$ embryos, $n = 21$ cells for anterior; $N = 3$ embryos, $n = 45$ cells for middle; $N = 3$ embryos, $n = 35$ cells for posterior. HC: $N = 2$ embryos, $n = 13$ cells for anterior; $N = 2$ embryos, $n = 25$ cells for middle; $N = 2$ embryos, $n = 17$ cells for posterior).
ns, not significant; $*p < 0.05$, $**p < 0.01$, $***p < 0.001$, $****p < 0.0001$.
Scale bar, 20 µm.

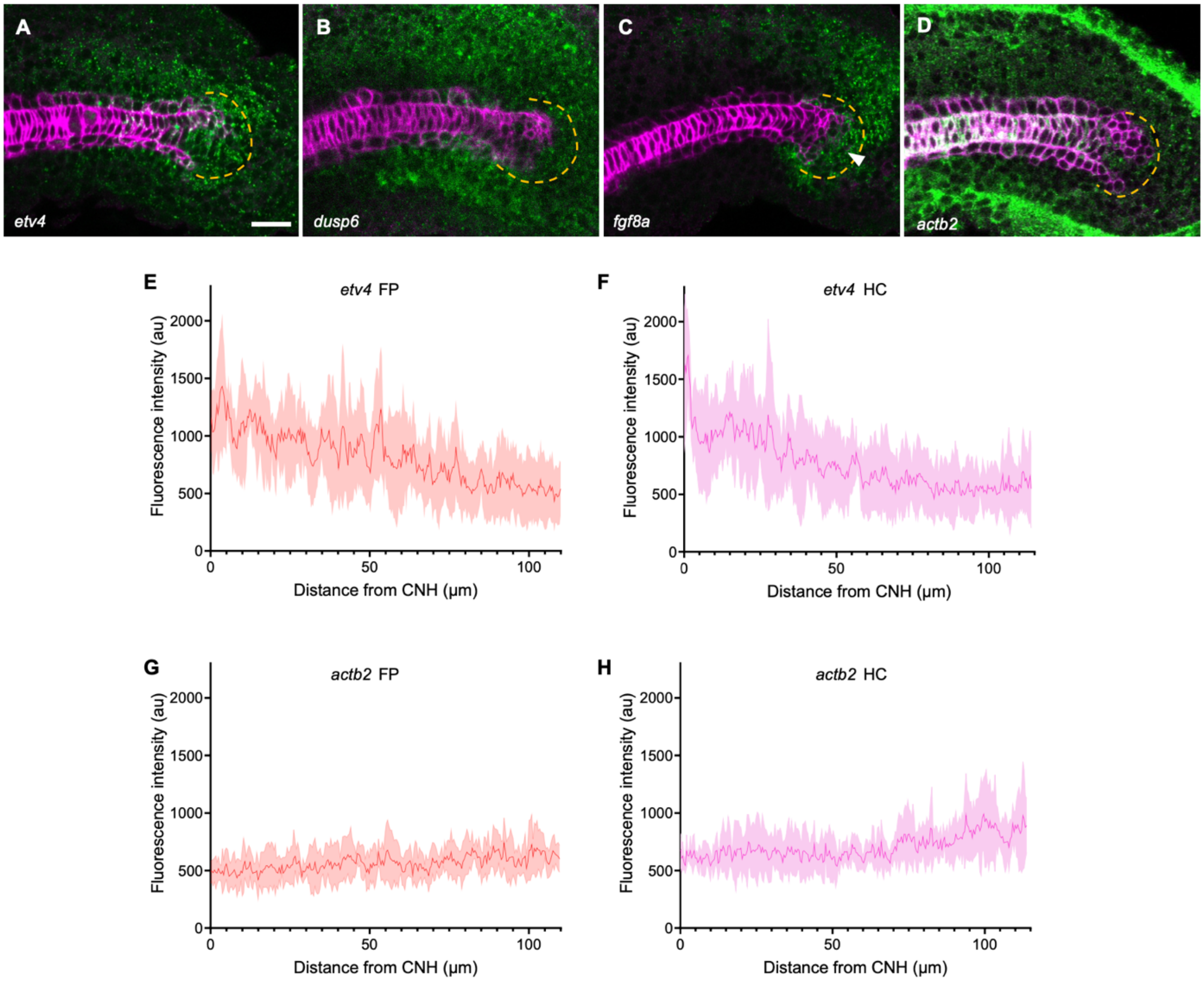

Fig. S4. FGF signaling activity in FP and HC cells along the anterior-posterior axis.

(**A, B**) RNA in situ hybridization chain reaction (HCR) for FGF downstream genes *etv4* (A) and *dusp6* (B), indicating the expression in FP and HC. Dashed lines delineate the CNH.
(**C**) RNA in situ HCR for *fgf8a* showing the expression in the CNH. Dashed line delineates the CNH. Arrowhead indicate a signal in the posterior end of notochord progenitor cells in the CNH.
(**D**) RNA in situ HCR for *actb2* showing ubiquitous expression in the tailbud including the midline tissues. Dashed line delineates the CNH.
(**E, F**) Plot profiles of fluorescent intensity of *etv4* HCR signals within FP (E, $n = 4$ embryos) and HC (F, $n = 4$ embryos) along the anterior-posterior axis from the CNH. The slopes indicate posterior-to-anterior gradients of the FGF signaling activity in FP and HC.
(**G**, **H**) Plot profiles of fluorescent intensity of *actb2* HCR signals within FP (G, $n = 5$ embryos) and HC (H, $n = 5$ embryos) along the anterior-posterior axis from the CNH.
Scale bar, 20 µm.

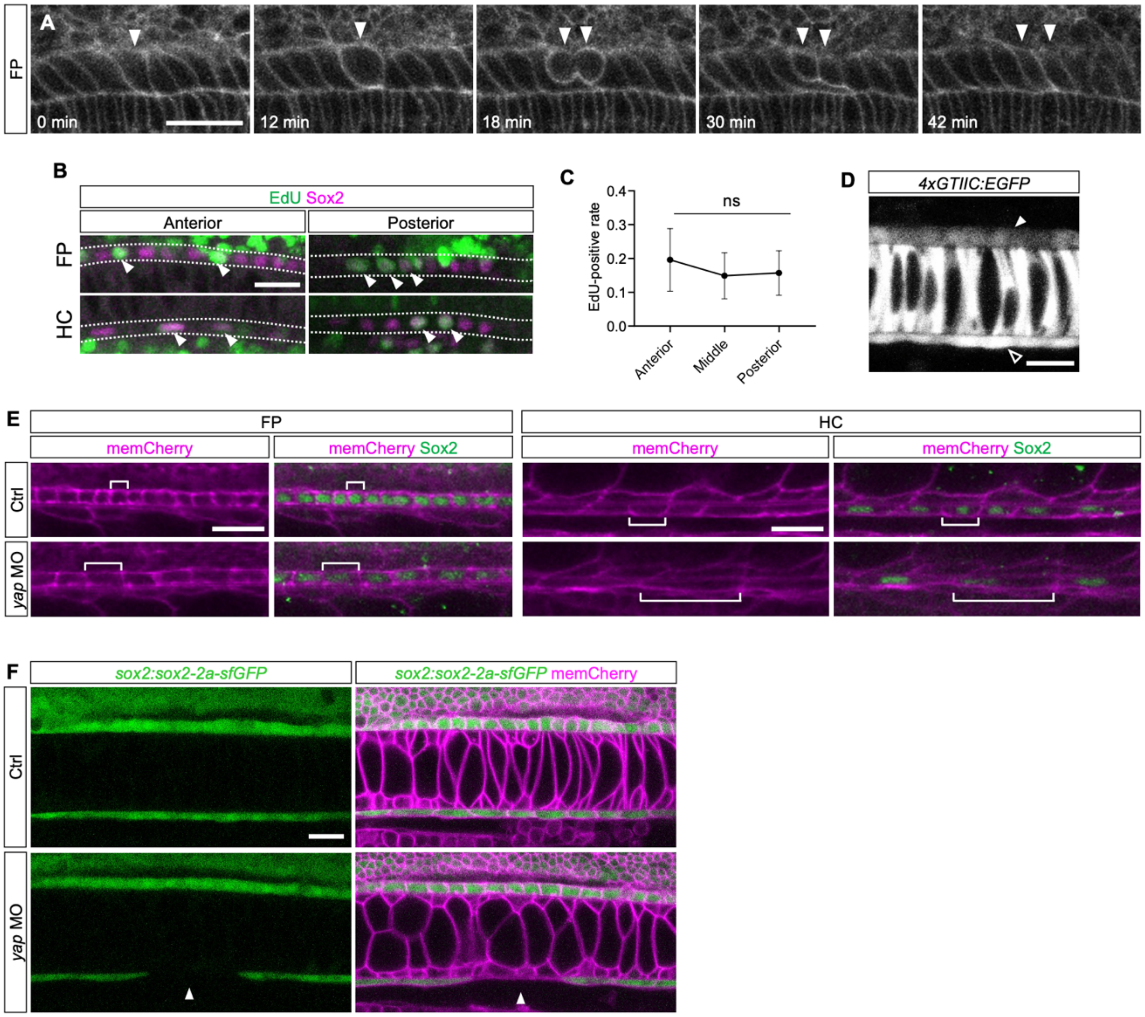

Fig. S5. Cell proliferation in the FP and HC, and *yap* morphant phenotype.

(**A**) Time-lapse images of FP cells in an *actb2:memCherry* embryo at 24 ss. Arrowheads indicate FP cells undergoing proliferation, with their daughter cells reintegrated into the tissue.
(**B**) EdU staining of 26-ss zebrafish embryos. FP and HC cells (delineated by dashed lines) are co-stained with anti-Sox2 antibody (magenta). EdU-positive FP and HC cells are indicated with arrowheads.
(**C**) Fractions of EdU-positive cells along the anterior-posterior axis of FP (n = 7 embryos).
(**D**) GFP expression of a *4xGTIIC:EGFP* reporter embryo. FP and HC cells are indicated by a filled arrowhead and an open arrowhead, respectively.
(**E**) FP and HC cells within 30-ss *actb2:memCherry* embryos injected with *yap* morpholino oligos, showing wider widths along the anterior-posterior axis compared to control embryos (brackets). Sox2 labels the nuclei of FP and HC cells (green).
(**F**) Lateral view of a 1-day *sox2:sox2-2a-sfGFP* embryo injected with *yap* morpholino oligos, showing a gap within the HC (arrowhead) while the control embryo has a continuous HC.
Anterior to the left.
Scale bars, 20 μm (A, B, D, E, F).

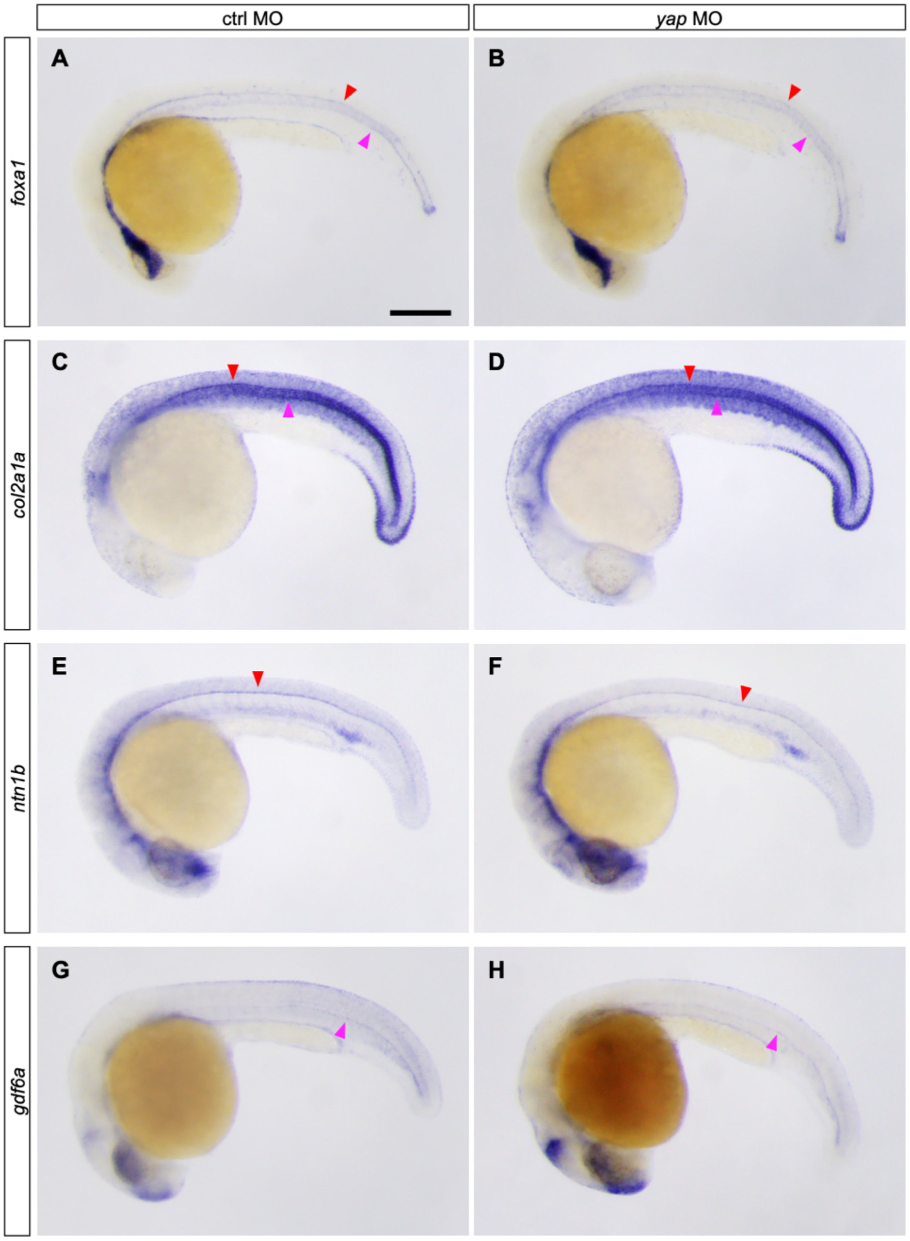

Fig. S6. Normal cell fate specification in *yap* morphant embryos.

In situ hybridization for FP and HC markers in embryos injected with control or *yap* morpholino oligos. *foxa1* (**A**, **B**) and *col2a1a* (**C**, **D**) are expressed in both FP and HC. *ntn1b* (**E**, **F**) is expressed in FP. *gdf6a* (**G**, **H**) is expressed in HC. Red and magenta arrowheads indicate FP and HC, respectively.

Scale bar, 200 µm.

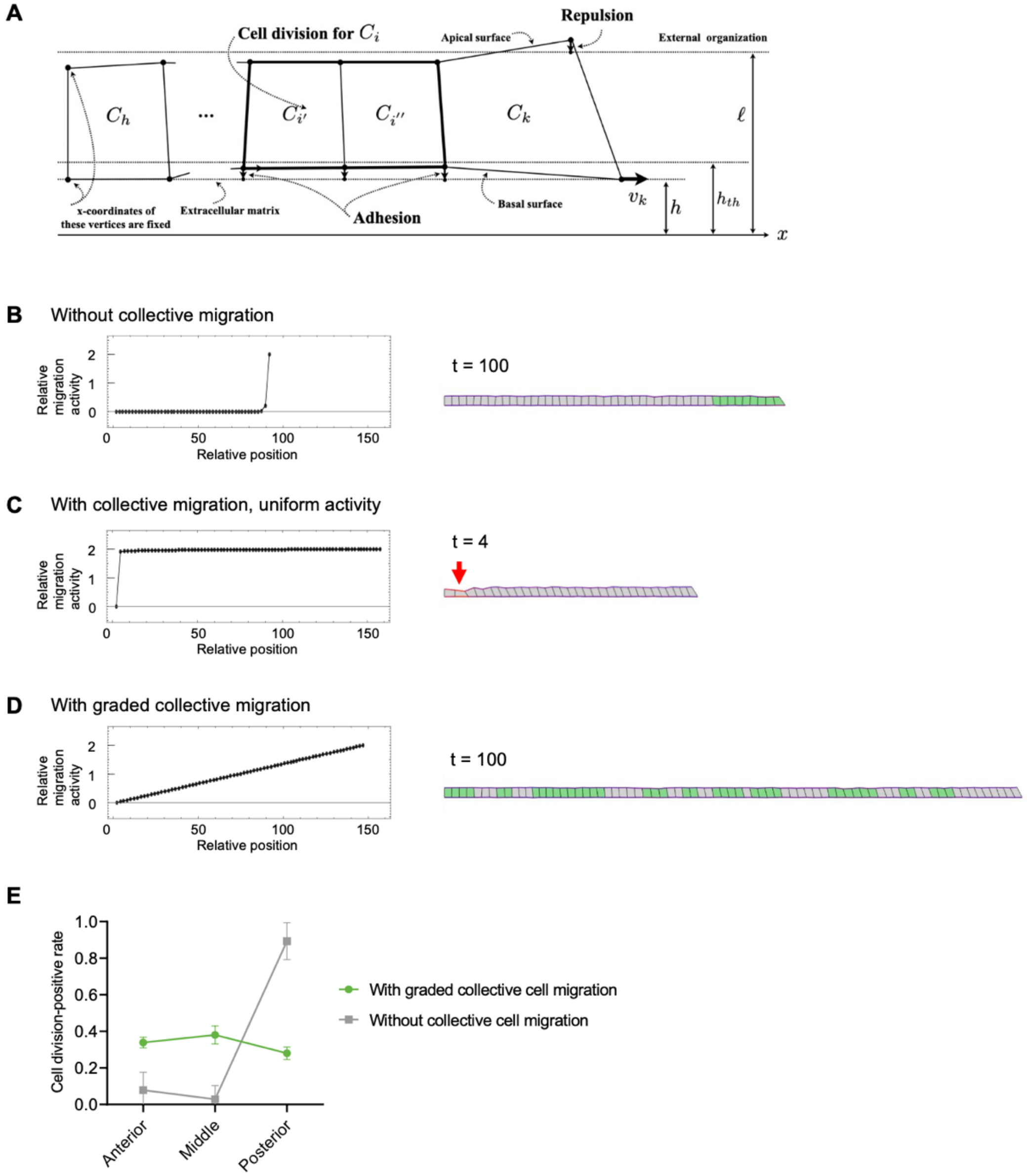

Fig. S7. Collective cell migration drives proper elongation of FP and HC.

(**A**) Schematic diagram of the vertex model. The cells C_h and C_k are the anterior-most and posterior-most cells, respectively. The anterior two vertices of the anterior-most cell C_h are fixed. The posterior vertex on the basal surface of the posterior-most cell C_k is subjected to the force v_k. For details, see 'Vertex model for tissue elongation' in Materials and Methods.

(**B-D**) Distribution of migration activity along the anterior-posterior axis (left) and simulated tissue elongation (right) of cells without collective migration (B), with spatially uniform activity of collective migration (C) or with graded collective migration (D). Cells that experienced mitoses from t = 50 onward are colored in green. Red arrow indicates a cell experiencing mechanical tensions exceeding the endurance threshold.

(**E**) Fractions of cells that experienced cell divisions without collective migration (grey, corresponding to B) or with graded collective migration (green, corresponding to D) along the anterior-posterior axis.

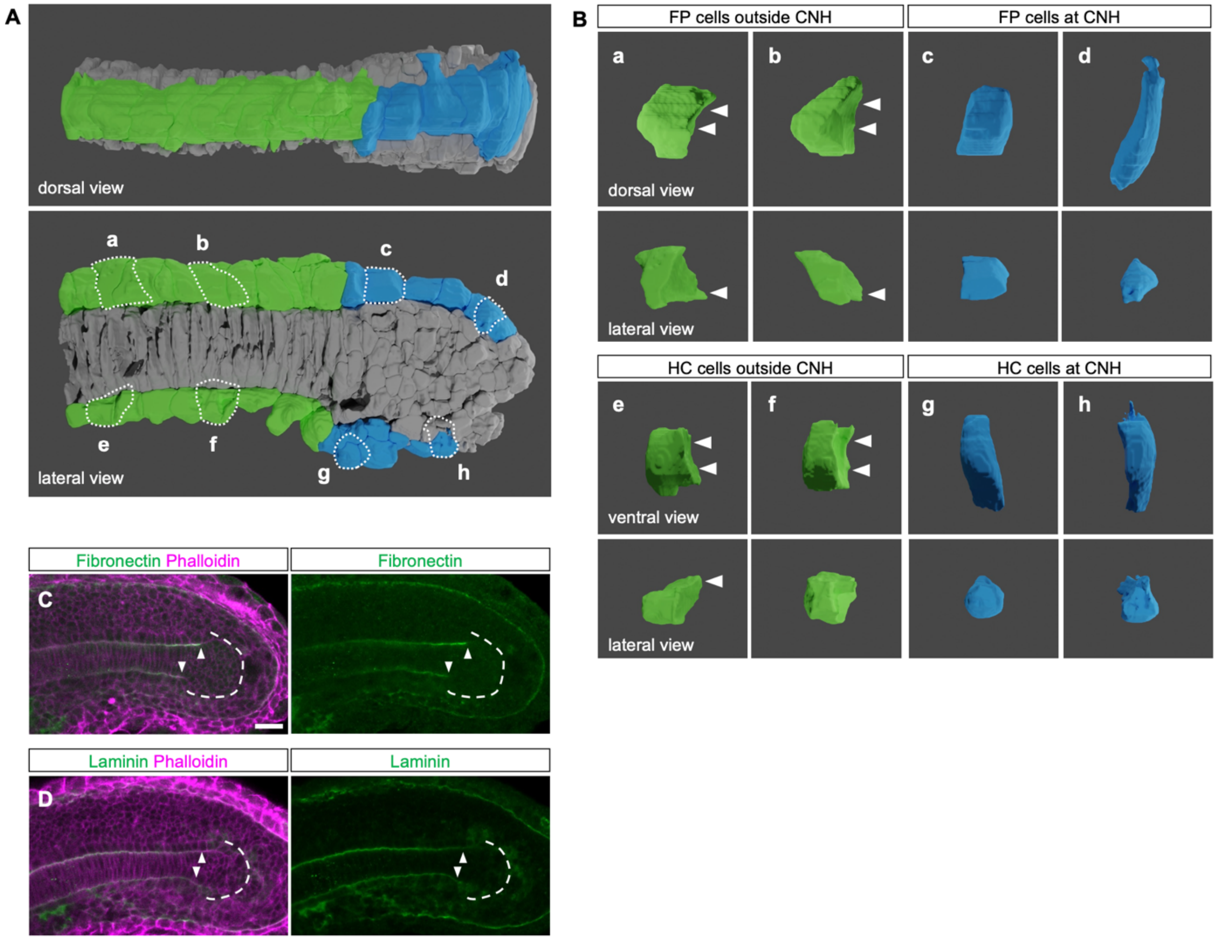

Fig. S8. The posterior end of the midline tissues.

(**A**) 3D-rendered view of the midline tissues in a *shha:memCherry* embryo at 22 ss. Green cells represent FP and HC cells, gray cells represent notochord cells and their progenitors, and blue cells indicate Sox2-positive cells surrounding the notochord progenitor cells.

(**B**) Magnified view of individual cells marked by dotted lines in (A). FP and HC cells located anterior to the CNH (a, b, e, f) exhibit posteriorly oriented basal protrusions (arrowheads). In contrast, cells surrounding the CNH (c, d, g, h) lack clear cellular protrusions and have elongated shapes along the medial-lateral axis.

(**C, D**) Fibronectin (C) and laminin (D) distribution in the tailbud. Posterior limits of the distribution within the midline tissues are indicated by arrowheads. Dashed lines delineate the CNH.

Scale bar, 20 µm.

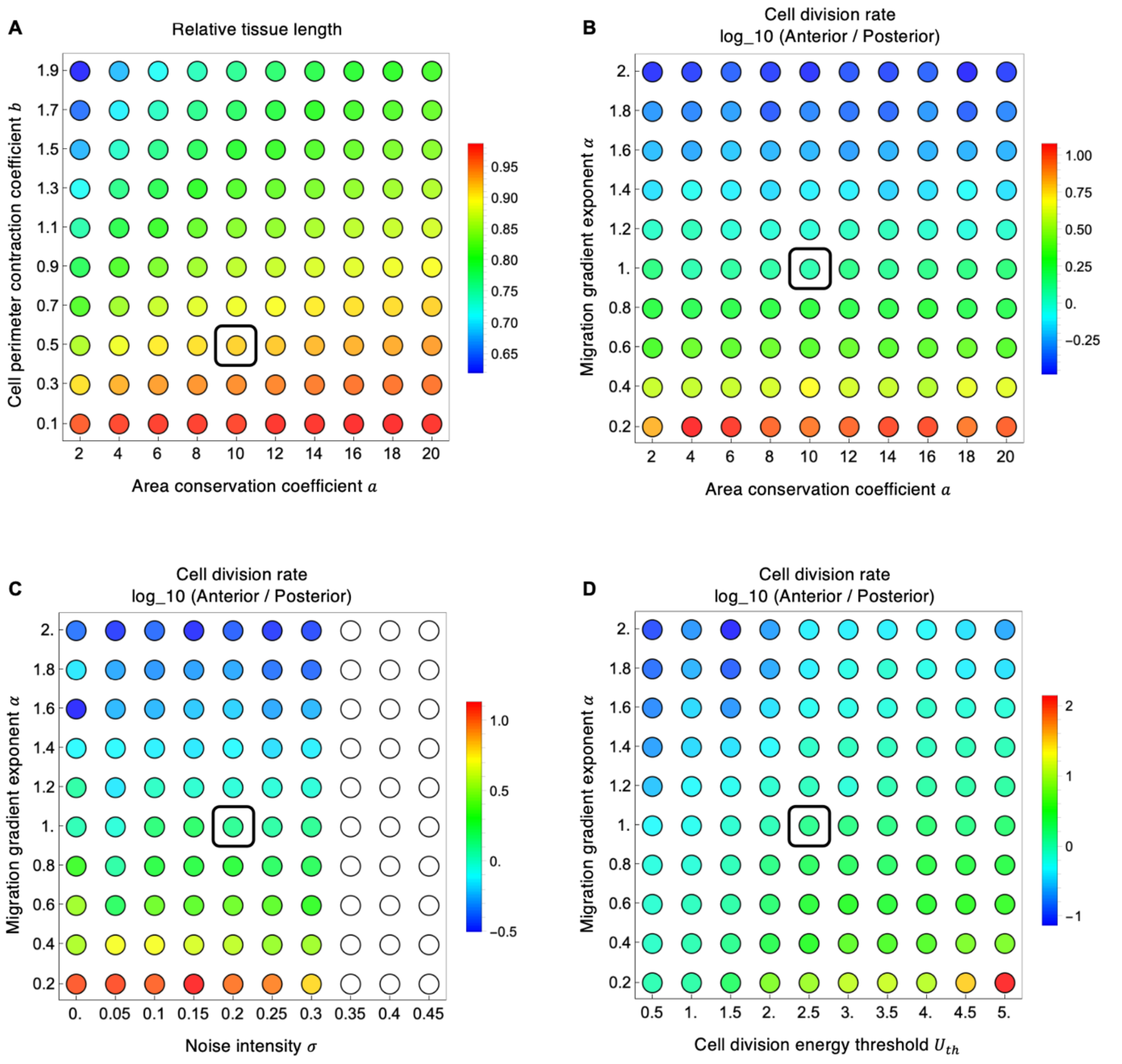

Fig. S9. Sensitivity of the computational model to changes in parameter values.

(**A**) Phase diagram of tissue length at $t = 0$ (when migration is absent, $v_{max} = 0$) as a function of area conservation coefficient a and perimeter constriction coefficient b. Tissue length is normalized to its initial value at $t = 0$ and indicated by color. Each point represents the average of seven trials. The outlined point corresponds to the parameter values used in the main simulation (see Table S1).

(**B**) Phase diagram of cell division rate during collective migration ($v_{max} = 2.0$, $\alpha = 1.0$) as a function of area conservation coefficient a and migration gradient exponent α. Cell division rate, calculated as the ratio of anterior to posterior cell divisions, is normalized using a logarithmic scale and indicated by color. Each point represents the average of seven trials. The outlined point corresponds to the parameter values used in the main simulation (see Table S1).

(**C**) Phase diagram of cell division rate as a function of noise intensity σ and migration gradient exponent α. Cell division rate is shown as in (B). Hollow circles indicate tissue collapse during the simulation. The outlined point corresponds to the parameter values used in the main simulation (see Table S1).
(**D**) Phase diagram of cell division rate as a function of the division energy threshold U_{th} and migration gradient exponent α. Cell division rate is shown as in (B). The outlined point corresponds to the parameter values used in the main simulation (see Table S1).

Supplementary Text. Sensitivity of the computational model to changes in parameter values.

In zebrafish embryos, experimental data are currently unavailable to directly parameterize the model. However, we carefully selected the critical parameter values for the following reasons and provide additional evidence to demonstrate the robustness of the model regarding these parameters.

1. **Parameters a and b:**
   Parameters a (cell area conservation) and b (cell perimeter constriction) were chosen to ensure that the tissue structure remains largely unchanged over time when cell division and migration are absent ($v_{max} = 0$). Fig. S9A illustrates the dependency of tissue length on a and b at $t = 100$ with $v_{max} = 0$. Tissue length increases with a but decreases with b. The values used in the main text (outlined in fig. S9A) yield a stable tissue length.
   Furthermore, parameters a and b have minimal impact on cell division dynamics. Fig. S9B shows the effect of varying a on cell division patterns under migratory conditions ($v_{max} = 2.0$, $\alpha = 1.0$). The dependency of unbiased cell division on a is negligible (fig. S9B, along the *x*-axis).

2. **Parameters c and d:**
   Similarly, parameters c (affinity of the basal surface to the ECM) and d (restorative force of the apical surface to its original height) were set to maintain tissue integrity. Smaller values of c and d led to tissue collapse, so we selected values that ensure stability during simulations.

3. **Spatial migration gradient exponent α:**
   The spatial distribution of migratory activity follows a power-law function, with the parameter α representing the exponent. When $\alpha = 1$, the gradient decreases linearly from posterior to anterior. For $\alpha > 1$, the gradient is convex downward, whereas for $\alpha < 1$, it is convex upward. Although cell division patterns vary with α, within the range of 0.85 to 1.5, the ratio of anterior-to-posterior division frequencies remains mostly consistent (within a 33% difference between anterior and posterior; fig. S9B).

4. **Noise intensity parameter σ:**
   The noise intensity parameter σ represents the strength of white noise applied to vertex forces. Fig. S9C shows the dependency of cell division rates on σ and α. Values of σ up to approximately 0.3 do not affect the simulation results. However, larger σ values destabilize tissue, leading to collapse (fig. S9C, hollow circles). The chosen $\sigma = 0.2$ in the main text ensures stability while retaining stochastic contributions to vertex dynamics.

5. **Elastic energy threshold U_{th} for cell division:**
   Fig. S9D shows the dependency of cell division rates on U_{th} and α. Lower U_{th} values cause posterior-biased cell division, while higher U_{th} values reduce the overall division frequency. The parameter value used in the main text yields an average division rate of 0.3 between time steps 50 and 100, which roughly corresponds to the in vivo EdU incorporation frequency (fig. S5C), supporting the validity of our choice.

In summary, our results indicate that fundamental parameters such as a (area conservation) and b (perimeter constriction) have limited impact on cell division. Instead, uniform cell division is primarily influenced by α, which governs the functional form of migration forces. Nevertheless, the model remains robust across a range of α values, supporting the validity of our conclusions regardless of specific parameter choices.

Notation	Value	Description
a	10.0	Coefficient for area conservation
b	0.5	Coefficient for perimeter contraction
c	100.0	Coefficient for adhesion between the basal surface and the ECM
d	100.0	Coefficient for apical surface repulsion
τ	1.0	Time constant for vertex dynamics
σ	0.2	Amplitude of white noise applied to vertex motion
η	1.0	Time constant for the external migration force
v_{max}	2.0	Maximum value of the external migration force
h	2.0	Position of the ECM along the y-axis
h_{th}	2.1	Adhesion threshold for basal vertices relative to the ECM
U_{th}	2.5	Threshold elastic energy required to initiate cell division
S_i	4.0	Time delay required for cell division after exceeding U_{th}

Table S1. Parameters used in the vertex model.

Movie S1. Time-lapse imaging of a tailbud during body axis elongation indicates a shearing among the midline tissues.

Time-lapse imaging of the tailbud of an *actb2:memCherry* zebrafish embryo starting at 22 ss. Arrowheads indicate neural (green), FP (red), notochord (blue) and HC (magenta) cells.

Movie S2. FP and HC cells collectively move posteriorly during body axis elongation.

Time-lapse imaging of the midline tissues of an *actb2:memCherry* zebrafish embryo at 22 ss. Arrowheads indicate FP (red) and HC (magenta) cells.

Movie S3. FP cells display transient cellular protrusions on the posterior side.

Time-lapse imaging of posterior FP cells in the tailbud of an *actb2:memCherry* (magenta) zebrafish embryo injected with Actin-Chromobody GFP (green) mRNA. Arrowheads indicate posteriorly polarized cellular protrusions.

Movie S4. FP cells at the posterior edge of an explant become squeezed during the culture.

Time-lapse imaging of a cultured explant showing that posterior FP cells adjacent to the edge gradually become thinner (arrowheads).

Movie S5. Daughter FP cells are reintegrated into the FP structure.

Time-lapse imaging of the midline tissues of an *actb2:memCherry* zebrafish embryo. Arrowheads indicate a cell division within the FP.

Movie S6. Simulation of tissue elongation under a condition where the only posterior-most cell is pulled posteriorly.

A two-dimensional vertex model representing a line of cells with the posterior-most cell mechanically pulled posteriorly. Cells that experienced mitoses from $t = 50$ onward are colored in green. Time is displayed on the top left.

Movie S7. Simulation of tissue elongation under a condition where all cells are given the same migratory activity.

A two-dimensional vertex model representing a line of cells with the same migratory activity posteriorly. Note that the cell membrane of one of the anterior cells experiences an excessive tensile force that exceeds the threshold at $t = 4$ (shown in red). Time is displayed on the top left.

Movie S8. Simulation of tissue elongation under a condition where the migratory activity of the cells is graded from posterior to anterior.

A two-dimensional vertex model representing a line of cells with the migratory activity graded from posterior to anterior. Cells that experienced mitoses from $t = 50$ onward are colored in green. Time is displayed on the top left.

Movie S9. Simulation of the elongation process of the three midline tissues under a condition where the notochord elongates faster without inter-tissue adhesion.

A two-dimensional vertex model representing the midline tissues without inter-tissue adhesion, where the notochord grows faster than FP and HC. The migratory activity is given to all cells of

FP and HC in a posterior-to-anterior gradient. Cells that experienced mitoses from $t = 50$ onward are colored in green. Time is displayed on the top left.

Movie S10. Simulation of the elongation process of the three midline tissues under a condition where the notochord elongates slower without inter-tissue adhesion.

A two-dimensional vertex model representing the midline tissues without inter-tissue adhesion, where the notochord grows slower than FP and HC. The migratory activity is given to all cells of FP and HC in a posterior-to-anterior gradient. Cells that experienced mitoses from $t = 50$ onward are colored in green. Time is displayed on the top left.

Movie S11. Simulation of the elongation process of the three midline tissues under a condition where the notochord elongates faster with inter-tissue adhesion.

A two-dimensional vertex model representing the midline tissues with inter-tissue adhesion at the posterior ends, where the notochord grows faster than FP and HC. The migratory activity is given to all cells of FP and HC in a posterior-to-anterior gradient. Cells that experienced mitoses from $t = 50$ onward are colored green. Time is shown on the top left.

Movie S12. Simulation of the elongation process of the three midline tissues under a condition where the notochord elongates slower with inter-tissue adhesion.

A two-dimensional vertex model representing the midline tissues with inter-tissue adhesion at the posterior ends, where the notochord grows slower than FP and HC. The migratory activity is given to all cells of FP and HC in a posterior-to-anterior gradient. Cells that experienced mitoses from $t = 50$ onward are colored green. Time is displayed on the top left.
